# Developing a FAIR data policy for chemical risk assessment and exploring future topics and stakeholder interests with large language models and data sciences

**DOI:** 10.1186/s12302-026-01472-y

**Published:** 2026-07-26

**Authors:** Rob Stierum, Gino Kalkman, Sabina Bijlsma, Jildau Bouwman, Susana Loureiro, Iseult Lynch, Barbara Magagna, Penny Nymark, Peter C. von der Ohe, Tessa E. Pronk, Erik Schultes, Karin Slater, Jan Theunis, Fred van de Brug

**Affiliations:** 1 TNO, Risk Analysis for Prevention, Innovation and Development, Princetonlaan 6, 3584 CB Utrecht, The Netherlands; 2 TNO, Microbiology and Systems Biology, Sylviusweg 71, 2333 BE Leiden, The Netherlands; 3 Universidade de Aveiro, Centre for Environmental and Marine Studies, Department of Biology, Applied Ecology and Ecotoxicology Laboratory, 3810-193 Aveiro, Portugal; 4 University of Birmingham, School of Geography, Earth and Environmental Sciences, Edgbaston, Birmingham, B15 2TT UK; 5 GO FAIR Foundation, Poortgebouw Noord, Rijnsburgerweg 10, 2333 AA Leiden, The Netherlands; 6 Karolinska Institutet, Institute of Environmental Medicine, Nobels Väg 13, 17177 Stockholm, Sweden; 7 UBA-German Environment Agency (Umweltbundesamt), Wörlitzer Platz 1, D-06844 Dessau-Roßlau, Germany; 8 KWR Water Research Institute, Groningenhaven 7, 3433 PE Nieuwegein, The Netherlands; 9 Flemish Institute for Technological Research (VITO), Environmental Intelligence Unit, Boeretang 200, 2400 Mol, Belgium; 10 TNO, Research Information Support, Anna van Buerenplein 1, 2595 DA Den Haag, The Netherlands

**Keywords:** Next generation chemical risk assessment, FAIR, Data policy, Large language models, Stakeholders, Partnership for the assessment of risks from chemicals (PARC)

## Abstract

**Background:**

The EU Partnership for the Assessment of Risk from Chemicals (PARC) develops novel methods for human health and environmental risk assessment (RA) for regulatory application. Large amounts of environmental monitoring, in vitro, in silico and other types of data are generated. To facilitate data reuse and information exchange within and between research and regulatory stakeholders, it is important to document and manage data. In 2016, the FAIR (Findable, Accessible, Interoperable and Reusable) data principles were introduced to foster reuse of data. To adopt FAIR within PARC, a PARC FAIR data policy (PFDP) was developed.

**Results:**

To determine PFDP topics, expert panel discussions were held, followed by review of 37 stakeholder documents issued by authorities in RA and environmental, health, (open) data sciences. This resulted in a taxonomy of 57 topics (“narrower term(s)” (NT(s), together with 15 “broader terms”), forming the basis of the PFDP. These included, aside FAIR, aspects specifically relevant for RA, such as GDPR, sensitive data, transparency and data valuation. Large Language Models (LLMs) were used to contrive novel terms for PFDP revisions. Seventy-eight novel terms were discovered by LLMs, compared to 57 NTs already identified by experts. Based on a frequency analysis within stakeholder documents, of all 135 terms (57 + 78) -further categorized under eight ChatGPT-derived “FAIR categories”- and subsequent cluster analyses (PCA, K-means), differences between stakeholders were identified. Organizations concerned with environmental and human chemical RA (e.g. European Chemicals Agency, European Food Safety Authority) could be distinguished from those focused on technical FAIR data issues and those involved in publicly funded research and open science.

**Conclusions:**

To foster environmental and human health research data for research purposes and adoption of research data in regulatory applications, a taxonomy of terms and FAIR data policy was developed. A frequency analysis of the taxonomy (augmented with LLM-derived) terms in stakeholder documents revealed differences between stakeholders, which may help identify trends e.g. the adoption of FAIR principles within and across sciences and regulatory domains for (NG) RA. The PFDP is designed to support data driven RA improvement within PARC but can equally contribute to FAIR data in other environmental RA research and regulatory communities.

**Supplementary Information:**

The online version contains supplementary material available at https://doi.org/10.1186/s12302-026-01472-y.

## Background

Following health and environmental incidents arising from chemical exposures [19–21] chemical risk assessment (RA) sciences have progressed rapidly, enabling the integration of environmental, animal and human data to identify chemical hazards, derive safe exposure limit values and compare these with environmental and human exposures to enable RA. Consequently, within Europe, European agencies (the European Environment Agency (EEA), the European Medicines Agency (EMA), the European Food Safety Authority (EFSA) and the European Chemical Agency (ECHA)) were established that assist in evaluating the risk to human and environmental health from chemicals utilized in different sectors.

Recently, the European Commission published the “Chemicals Strategy for Sustainability. Towards a Toxic-Free Environment” [8]. The goals in the Strategy are highly challenging, as there are many chemicals released to/found in the environment for which RA is absent or at best incomplete. Next-Generation Risk Assessment (NGRA) approaches are being developed to accelerate risk assessment by reducing the reliance on long-term in vivo testing of individual chemicals and instead using non-animal methods or new approach methodologies (NAMs). These include, among others, in silico and in vitro cell systems and non-targeted analyses to identify emerging environmental contaminants. To facilitate the incorporation of emerging NGRA methods into regulatory frameworks, the Partnership for the Assessment of Risks from Chemicals (PARC) project was launched [18]. PARC is of unparalleled scale, and joins ~ 200 European health and safety agencies and research organizations.

A wide range of data types are being generated within PARC, spanning human and environmental biomonitoring, toxicology and RA. These data need to be curated and managed in a sustainable infrastructure to facilitate transparent data integration, preservation and reuse. Towards this end, the PARC Chemical Risk Assessment Data Hub [25] is in development which will serve as a metadata registry and provide links to the distributed PARC data (stored in PARC-approved domain-specific databases). Effective data curation prevents the duplication of data generation, ethically unfavorable when involving animals or humans, thereby avoiding the unnecessary depletion of research funds. Furthermore, large scale data reuse can improve RA models, while decreasing uncertainty [17]. Examples are the improvement of quantitative exposure response relations [27], systems toxicology to predict PFAS effects on cholesterol homeostasis [30] and text mining to identify key characteristics of carcinogens [28]. At the same time, experience from these studies showed that finding, retrieving, and understanding the (meta) data was very challenging, in some cases requiring emails to authors to request missing information, and thus that reusing the data was highly inefficient.

In addition, the availability of high-quality data is considered to be pivotal to enable implementation of Safe and Sustainable by Design approaches [16]. For all these reasons, the findability, exchange and reuse of research data for RA purposes should be facilitated.

**Fig. 1:**
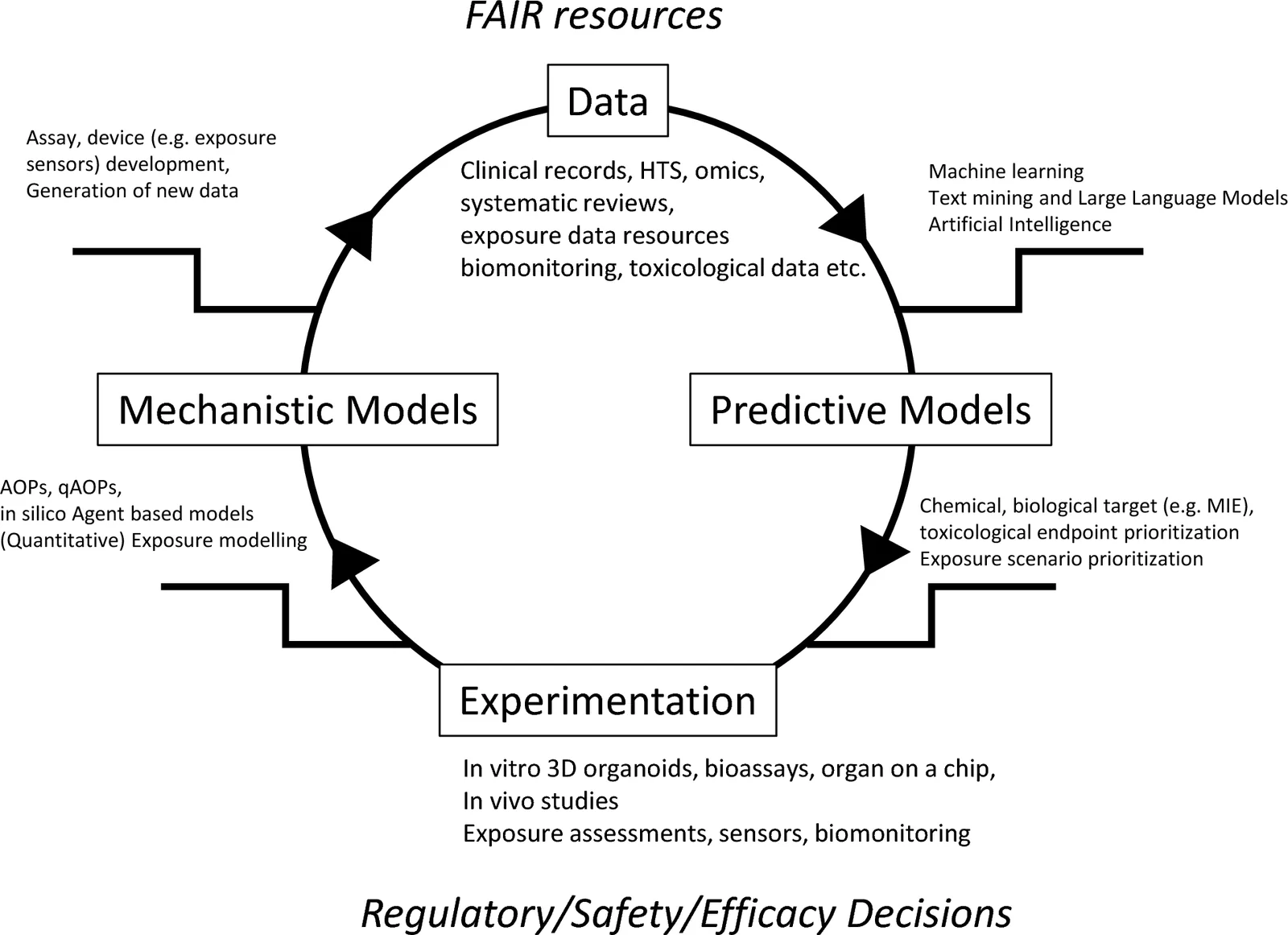
Iterative data reuse for improving chemical RA. The reuse of data, facilitated by data technologies leads to predictive models, which in turn can propose novel experimentation strategies in turn leading to creation of new mechanistic models, assays and data for reducing model uncertainty. Ultimately, this can improve chemical RA. The application of FAIR principles can improve the findability, accessibility, interoperability and reusability of research data (and models) essential to foster the iterative data reuse cycling to improve chemical RA. Figure 1 is inspired by an original conception by Dr. Nicole Kleinstreuer, NICEATM, USA [17] and a talk provided by Dr. Penny Nymark [22]. *AOP* Adverse Outcome Pathway, *qAOP* quantitative AOP, *HTS* High-throughput Screening, *MIE* Molecular Initiative Event (start of an AOP)

In 2016, the FAIR (Findable, Accessible, Interoperable and Reusable) principles were proposed [31]. These principles are intended to enhance the reusability of data by researchers as well as computational systems, and to speed up (interdisciplinary) data integration, of particular relevance to PARC (Fig. 1).

To stimulate this process, we describe here the development of the PARC FAIR Data Policy (PFDP). A version of record is available via Zenodo [29] and extensive metadata for the PFDP is available from FAIRsharing.org [11]. Earlier (and ongoing) research addressed the importance of FAIR within specific areas of RA sciences. Examples are in nanosafety [15], human biomonitoring [33], exposome research [12] and environmental health and remediation [14]. The present work extends these findings by developing an overarching PFDP, applicable to all RA sciences, required for the comprehensive data driven innovation of RA.

Towards this end, expert panel discussions and analyses of (amongst other things regulatory) documents from a wide range of stakeholders in the fields of health, FAIR data, open science and chemical RA were organized to retrieve relevant topics (terms) to cover in the PFDP. During this process it was recognized that for chemical risk assessment additional terms proposed by experts and stakeholder documents beyond those in the FAIR principles are relevant to guide data management practices in PARC.

Additionally, we hypothesized that Large Language Models (LLMs) could be employed to improve future revisions of the policy. Indeed, we observed confirmation of terms already proposed by expert knowledge and stakeholder documents but also discovered novel terms that can complement these. Finally, multivariate analyses performed on the frequencies of the expert and LLM derived terms, within the stakeholder documents, successfully suggested the existence of differences among stakeholders in the field of health, data, open science and chemical RA in terms of their current focus.

## Methods

**Fig. 2:**
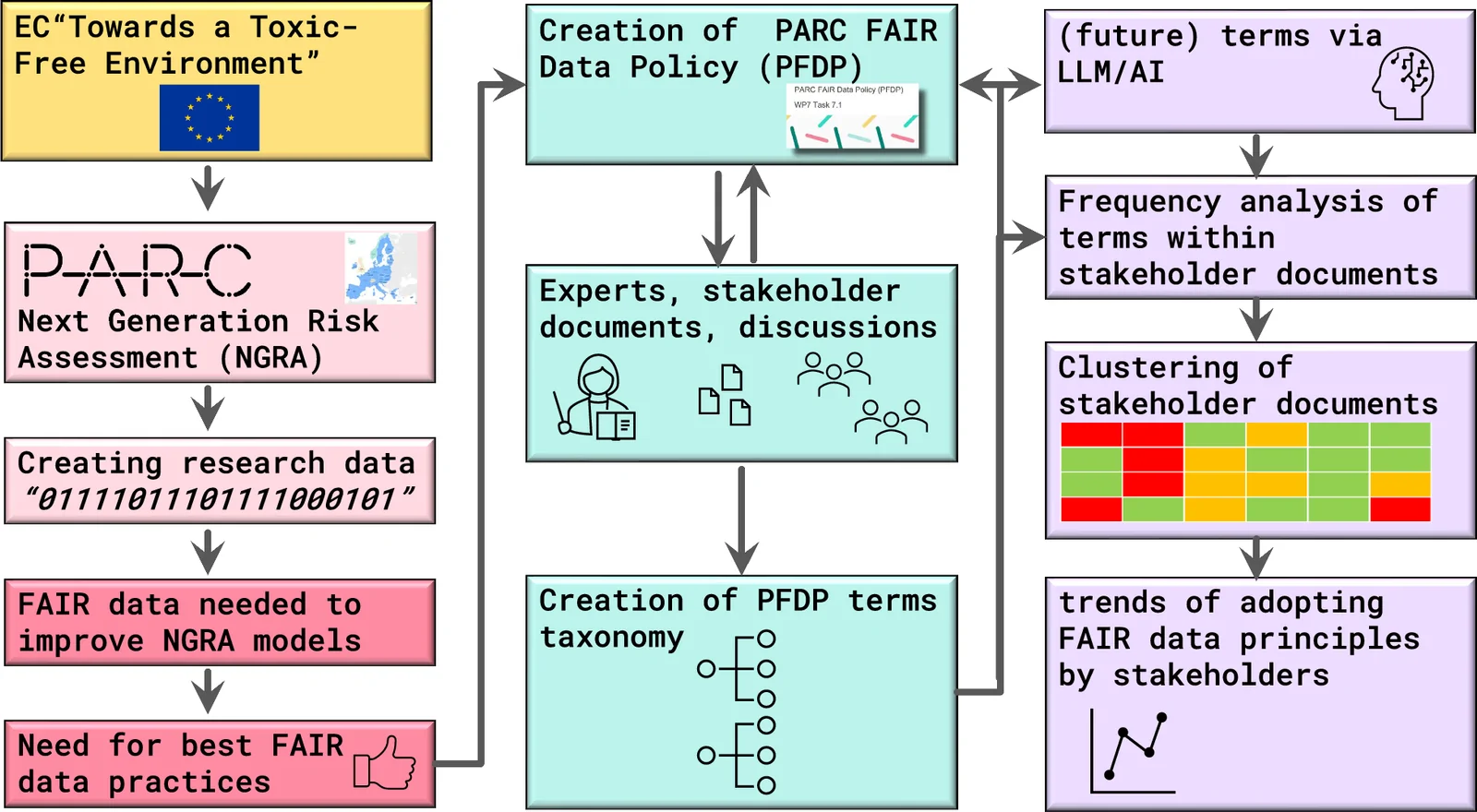
Overview of the methodology. The reasoning and process for the development of a FAIR taxonomy and PFDP for chemical risk assessment are outlined, together with an exploration of novel topics using LLM/AI and stakeholder document analysis

Figure 2 provides an overview of the need for and methodology used to develop a FAIR taxonomy and PFDP for chemical risk assessment, together with an exploration of novel topics using LLM/AI and stakeholder document analysis. Each of the different steps are described in detail below.

### Creation of the PFDP

**Fig. 3:**
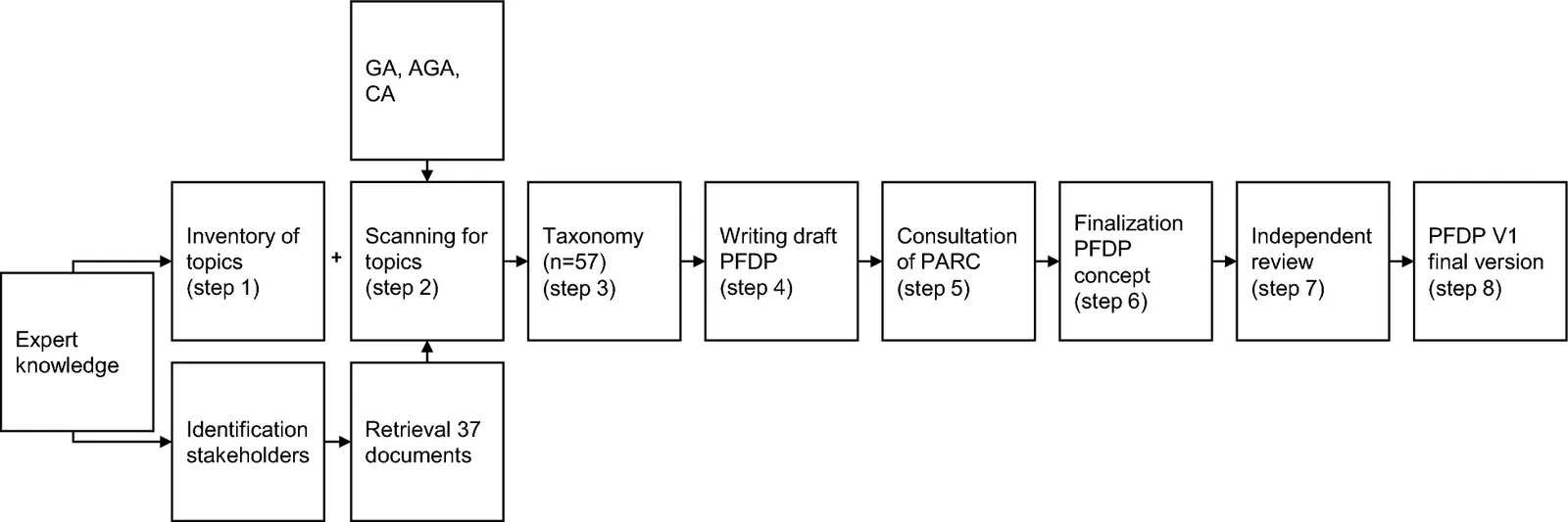
The process of the development of the PFDP. The collection of topics, resulting from step 1 (expert knowledge) and step 2 (stakeholder documents and the PARC Grant Agreement (GA), Annotated Grant Agreement (AGA) and the Consortium Agreement (CA)) served to finalize topics within a hierarchy of terms (step 3 ). This taxonomy/hierarchy consisted of Narrower Terms (NTs) and Broader Terms (BTs) and served as the backbone to write the draft version of the PFDP (step 4). The policy text was then further enriched by the input from the online consultation rounds of the PARC community in step 5. The concept version of the policy (step 6) was then reviewed by two reviewers not previously involved in the drafting of the policy (step 7). The then updated version of the PFDP was checked with the PARC coordinating team and subsequently submitted to the EU Portal (step 8)

In detail, the creation of the first draft PFDP involved various steps (Fig. 3). To identify topics spanning FAIR data principles and environmental and human chemical RA, a panel of 50 experts (Supplementary Information Table S1, footnote “1”) gathered on 6–7 October 2022 (20 in Brno, Czech Republic, 30 via Microsoft Teams) to compose and discuss a first list of topics (step 1, Fig. 3).

To enhance this list, possible relevant organisations were identified by knowledge exchange and discussion between the 5 PARC scientists (amongst the co-authors, expertise in Supplementary Information Table S2, footnote “1”). To enable coverage across different topics, these stakeholders were identified from EU regulatory agencies, other EU initiatives, FAIR initiatives/FAIR data domains, open science, repositories, international organisations, academics and publishers. Next, organisational websites were identified via Google searches. The corresponding landing pages and their subpages were then reviewed to locate potentially relevant reports, position papers and publications. These are indicated in detail in “Supplementary Information Table S2”and “Supplementary Information Stakeholder Documents.pdf”. Where available, the website’s internal search function was used with the terms “FAIR”, “data policy”, and “data management” to retrieve additional information.

Next, these 37 identified documents were scanned for relevant topics (step 2, Fig. 3) from which text snippets were extracted. The final selection of the topics was made by one of the 5 scientists, followed by a final reflection by the other 4.

Next, a taxonomy was developed (step 3, Fig. 3). Towards this, stakeholder document derived topics were categorized into a two-layer hierarchy/taxonomy of “Narrower Term(s)” (NTs) and “Broader Term(s)” (BT(s)). First, manually extracted topics from text snippets with similarity/commonality were brought together into 57 separate concepts, to reduce redundancy and overlap. This was done within the group of 5 scientists, in which expert knowledge from two scientists with backgrounds in toxicology/risk assessment and semantic interoperability/data was first employed to propose similar and common topics. Next, online sessions amongst all 5 scientists were held to discuss the outcome of this process and reach consensus on the final list of 57 concepts.

These are referred to as NTs. Subsequently, in relation to these 57 NTs, 15 so called BTs were proposed. These are at higher level in the hierarchy and serve to combine multiple related NTs into a broader category. The naming of these information layers (NT, BT) within this taxonomy was derived from a taxonomy glossary developed by Taxonomy Strategies (19). The development of the taxonomy was based upon expert judgement using the information within the topics/text snippets from the stakeholder documents and further informed by the EU Annotated Grant Agreement [9] and the PARC project Grant and Consortium Agreements. Specifically, the expert knowledge of all 5 scientists was used to propose 15 broader terms (BTs) and reach consensus on distributing the 57 narrow terms (NTs) across these BTs. To avoid bias, when available, formalized term definitions were retrieved (and included in the PFDP) from stakeholder documents, websites and other international sources (e.g. [3, 6, 13, 24, 32]). The taxonomy served to structure the writing of the draft PFDP (step 4, Fig. 3). This was done by a “writing team”, consisting of the 5 PARC scientists mentioned, with 5 additional scientists with expertise on FAIR data/RA.

As stated in the PARC Project Grant Agreement: “In line with Article 8.6 of the Consortium Agreement, each participant of PARC will be required to comply with PARC FAIR Data Policy…”. In light of this requirement, and to promote the ultimate acceptance of and adherence to the PFDP, efforts were undertaking to build consensus and foster support already during its development. Specifically, the PARC community was consulted on the draft PFDP (step 5, Fig. 3). Microsoft Forms were used to approach ~ 2000 individuals from ~ 200 universities, research institutes, health (care) organisations, EU agencies to collect input. Secondly, topics were selected for which the need for a more in-depth discussion with a smaller subset of the PARC community was anticipated. These were: “data governance”; “management of sensitive data”; “open data (including embargo/release)”; “data quality”; “FAIR data together with ambitions and levels of FAIRness and implementation roadmaps”. Next, in Microsoft Teams sessions, a subset of stakeholders and PARC members (332 individuals) (Supplementary Information Table S3) were invited to discuss these topics, to help improve the PFDP (step 6). A final review by two independent scientists within PARC, not involved in drafting the PFDP, (with expertise in ecotoxicology, nanomaterials, data management) (step 7) was performed after which the PFDP was submitted to the EU (project deliverable D7.2) (step 8).

### Enhancement of the original topics using LLMs

**Fig. 4:**
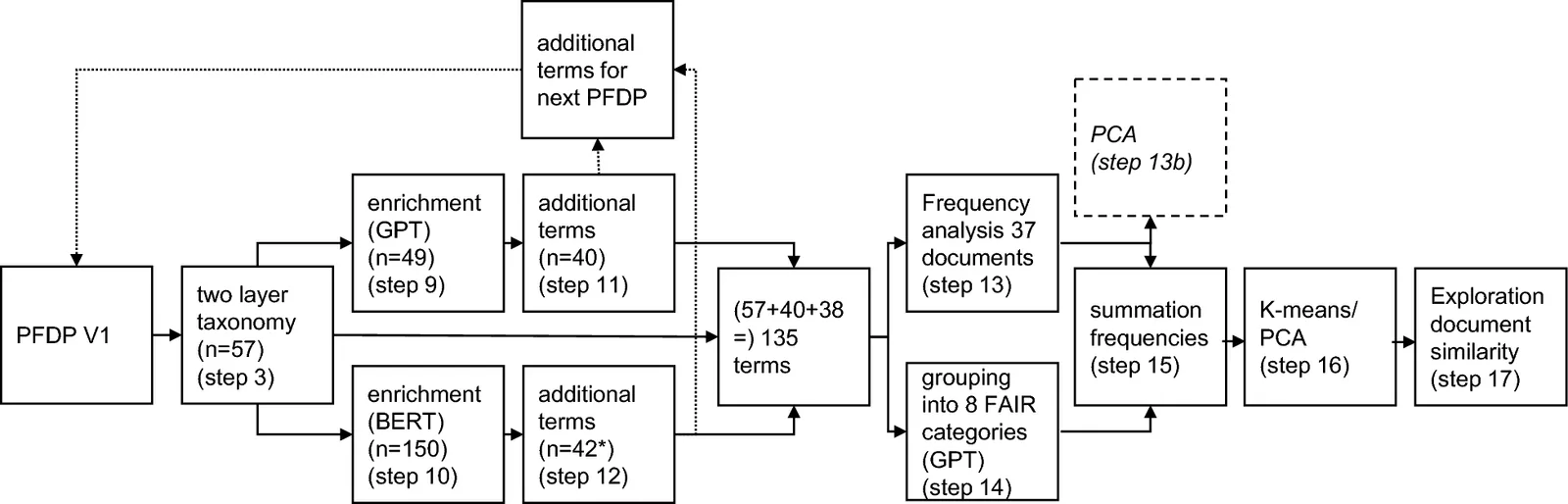
Overview of the LLM supported methodology for PFDP term enrichment followed by an explorative analysis of differences/similarities across documents obtained from different stakeholders. The 57 NTs [3] were used as input for LLM enrichment [9, 10], the outcome of which was expert evaluated [11, 12] before combination with terms derived from the taxonomy (yielding a total of 135 terms). The frequency of terms was assessed within the 37 stakeholder documents [13], and subjected to PCA [13b]). These 135 terms were also grouped in a FAIR data oriented manner, using GPT, resulting in 8 overarching “FAIR categories” [14]. For each of these “FAIR categories”, the normalized frequency per cluster was calculated [15] and clustered [16], to identify possible similarities between stakeholder documents [17]. *42 terms, with 4 identical terms also discovered by GPT, leaving 38 unique terms

We explored whether the original taxonomy could be enhanced by LLMs (Fig. 4). Updates of the PFDP are foreseen, as PARC is a 7-year project, and LLMs may help propose novel topics for these updates. We followed two approaches. OpenAI’s GPT model (version 3.5-turbo) (“GPT”) [23] (step 9, Fig. 4) was used with the 57 NTs (Table 1) as input, asking it to return a list of maximum 50 new terms being ‘similar to’ (i.e. sharing semantic properties with) the input terms. The query was “give suggestions for 50 relevant terms being similar to original terms” (with the original terms being the list of 57 terms, added thereafter). GPT is a generative LLM, the aim of which is to generate language in interaction with the user. GPT prompts the LLM to generate a response to a specific query from the user.

**Table 1:** Taxonomy of terms

Broader terms	Narrower terms
Accessible	Data access
	Efficiency
Applicable laws and regulations (general)^1^	Applicable laws and regulations (laws, contracts, funder requirements, policies, guidelines, (combined/aligned with the EU Common Open Data Platform, ECHA, Horizon Europe, OECD, Science Europe).)^1^
Archiving	Preservation
	Retention and deletion
Data governance (general)^1^	Aim of the policy^2^
	Data depositor^3^
	Data governance (process, Board, committee etc.)^1^
	Data valuation
	Ownership
	Roles and responsibilities
	Stakeholder needs^4^
	Warranty and liability
Data management (general)^1^	Data curation
	Data integrity
	Data management (tool, process)^1^
	Data quality
	DMP
	Documentation
	Research integrity
Data policy	Other guidelines/frameworks
	Policy implementation
	Policy review
	Scope and aim of the data policy
Data security	Data security
FAIR	FAIR
Findable	(Meta)Data^5^
	(Meta)Data schemas
	(Meta)Data sharing
	Discoverability
	Persistent identifiers
	Researcher identifiers
Intellectual property (general)^1^	Data citation^6^
	Intellectual property (e.g. forms of IP (patent., invention etc.)
Interoperable	Interoperability
	legal interoperability
	Organisational interoperability
	Semantic interoperability
	Technical interoperability
Repositories	Repositories
	Resource accreditation
	Service-level-agreements
	Sustainability
Reuseable	Data reuse
	Licenses
	Provenance
Sensitive data	Citizen science^7^
	GDPR
	Sensitive data (businesses)
	Sensitive data (personal)
Transparency and openness	Data availability statement
	Embargo^8^
	Open access
	Open data
	Open science
	Openness
	Transparency

The extracted topics from stakeholder documents were brought together into 57 separate concepts, referred to as Narrower Term(s) (NT(s)), to reduce redundancy. In relation to these, 15 Broader Term(s) (BTs) were proposed to serve the combination of multiple related NTs into a broader category. The development of the taxonomy was based upon expert judgement using the information within the topics/text snippets from stakeholder documents and informed by the EU Annotated Grant Agreement (20), and the PARC project Grant and Consortium Agreements

^1^After the peer review process of this Article, information in between parenthesis was added for further clarification to the readers. As the taxonomy was created via a careful process as described under the Methods section, these additions should not be considered as a change of the results/outcome of this process

^2^Aim of the policy” was categorized under “data governance” as policies are employed within data governance processes. “Aim of the policy” was included separately as the taxonomy served the development of the PARC specific FAIR data policy (PFDP), for which there was a necessity that the “aim of the policy” was described (https://doi.org/10.5281/zenodo.15005810, page 11)

^3^ “data depositor” (NT), this is a separate term under “data governance” (BT). The reason is that PARC wants to promote the reuse of data, and therefore data submitting (depositing) to repositories is an important step in the governance of data, which needed to be emphasized, also for creation of he PARC FAIR data Policy. The number of published/deposited data sets is a KPI for PARC. Therefore, the role of data depositor/submitter under data governance was essential to emphasize

^4^ “stakeholder needs” is under “data governance”. The reason for this is that the organisation of data governance can involve for example a data governance board and a data office. Stakeholder needs are taken on board in these organisational entities.”

^5^During the peer-review process of this Article, it was suggested that “metadata” could also be positioned under BT “FAIR”. We acknowledge that this is a valid alternative interpretation; however, to reflect PARC’s initial priority of ensuring metadata findability, we have retained its placement under ‘Findable’.”

^6^In this context, data citation is used to track novelty of a research result for patenting for example (as alternative interpretation, this term could also have been under “Reusable”)

^7^NT “citizen science” was added to BT “sensitive data”, aside the NT “sensitive data (personal)”. This to emphasize separately that (scientific) data created by citizens themselves may be sensitive as well. The reason for creating this separate NT term is that within PARC, the term “sensitive data (personal)” may primarily be associated with human biomonitoring data, rather than to citizen science-derived data. The addition of NT “citizen science” under BT “sensitive data” is to emphasize that PARC scientists should realize that data generated via “citizen science” may also be sensitive data

^8^While Embargo (period) might appear to be the opposite of “Open”, PARC follows the principle of “As open as possible, as closed as needed” and thus clarity around any embargo periods, and the justification for these, supports transparency and openness

In addition, Google’s BERT base model [5] (“BERT”) (step 10, Fig. 4) was used in combination with the stakeholder documents, again using the 57 NTs as input. BERT is a pre-trained LLM that encodes the semantic properties of language (words, sentences, etc.) and can be finetuned on any type of natural language processing (NLP) task which can be used to convert words into so called embeddings: vectors that describe the semantic properties of words. Next, the embeddings generated were averaged into a signature embedding, representing a summary of the semantic features of all terms in the original list of 57 NTs. Then, we identified candidate terms for enrichment by employing the Python Spacy library to extract all noun phrases (to extract the most important information from the text as nouns are the fundaments of text) from the 37 stakeholder documents. Again, we used the BERT model to generate embeddings for these noun phrases too, and subsequently compared the resulting embeddings to the signature embedding generated for the original term list. Finally, both sets of terms (from GPT and BERT) were subjected to deduplication and evaluated to exclude out-of-scope concepts or ambiguous terms. The outcome was compared to NT (and BT) terms already derived in the expert meeting and taxonomy to judge the novelty.

### Frequency analysis within stakeholder documents of terms derived from the taxonomy and LLM modelling

The frequency of terms derived from the taxonomy and LLM was determined within the stakeholder documents, for two purposes. The first purpose was to identify if LLM (and taxonomy) derived terms could be identified within the stakeholder documents. This was to signify if LLM can discover (novel) terms relevant to stakeholders. To this end, for each term, a normalized frequency analysis (step 13, Fig. 4) was executed by dividing the number of times a particular term was found in a stakeholder document, by the total number of occurrences of those terms, out of the possible maximum of 135 terms, in that particular document.

The second purpose was to identify clusters amongst the 37 stakeholder documents, with similar term frequencies, in order to explore possible similarities/differences in how different stakeholders describe aspects of (FAIR) data for data management/open science in general and specifically for chemical RA.

The outcome of the frequency analysis was used as input into principal component analysis (PCA) and K-means clustering (step 13b, Fig. 4), using Python’s scikit-learn package. For K-means clustering, we calculated the silhouette score to determine the optimal number of clusters. This metric can be used to measure the similarity of each sample in a cluster to its own cluster, compared to other clusters. The higher the score, the better defined the clusters are.

The initial PCA analysis after autoscaling of the data was not informative as principal components (PC) 1 to 3 explained only ~ 25% of the variance in the dataset. Therefore, it was decided not to further evaluate the outcome as the data appeared to be too noisy. To resolve this, a FAIR-data oriented grouping of the 135 terms under a smaller number of overarching “FAIR categories” (step 14, Fig. 4) was performed by asking GPT, without further imposing any requirements as to how the “”FAIR categories” should look or what their size should be, etc. This resulted in the 135 terms being divided over 8 overarching “FAIR Categories” (Supplementary Information Table S4), the overall adequacy of which was verified. For each of these categories, we summed the frequencies of the underlying terms in the 37 documents and normalized the total count in the same way as the counts for the 135 individual terms were normalized (step 15, Fig. 4). This resulted in a normalized frequency per overarching “FAIR category”, per document. These were subjected to PCA and K-means clustering (step 16, Fig. 4), followed by an explorative interpretation (step 17, Fig. 4).

## Results

### Creation of the PFDP

The two-day expert session to start the development of the PFPD, yielded a list of possible relevant topics (Supplementary Information Table S1). These covered:

- Technical aspects of data, e.g. “data formats”, “raw data”, “processed data”;
- FAIR criteria: “FAIR identifiers” and “level of FAIRness”, “Minimum FAIR criteria for summary data”;
- Metadata and standards”FAIR metadata”; “community standards”; “summary data”;
- Reuse of data “Data need an appropriate license”; “Share and reuse for other (EU) projects”,
- Scope of the policy: “Scope of the PFDP (e.g. software)”.

This list was further enhanced by exploring stakeholder documents (Supplementary Information Table S2). Documents were identified and screened for relevant text snippets, (Supplementary Information Table S2, column “prioritized content”). The hyperlinks are provided in this Table, the original documents/websites were also downloaded as PDF (“Supplementary Information Stakeholder Documents.pdf”).

Based upon the expert meeting and prioritized stakeholder content, topics were further selected and defined into a two-layer hierarchy of terms (taxonomy) (Table 1).

**Fig. 5:**
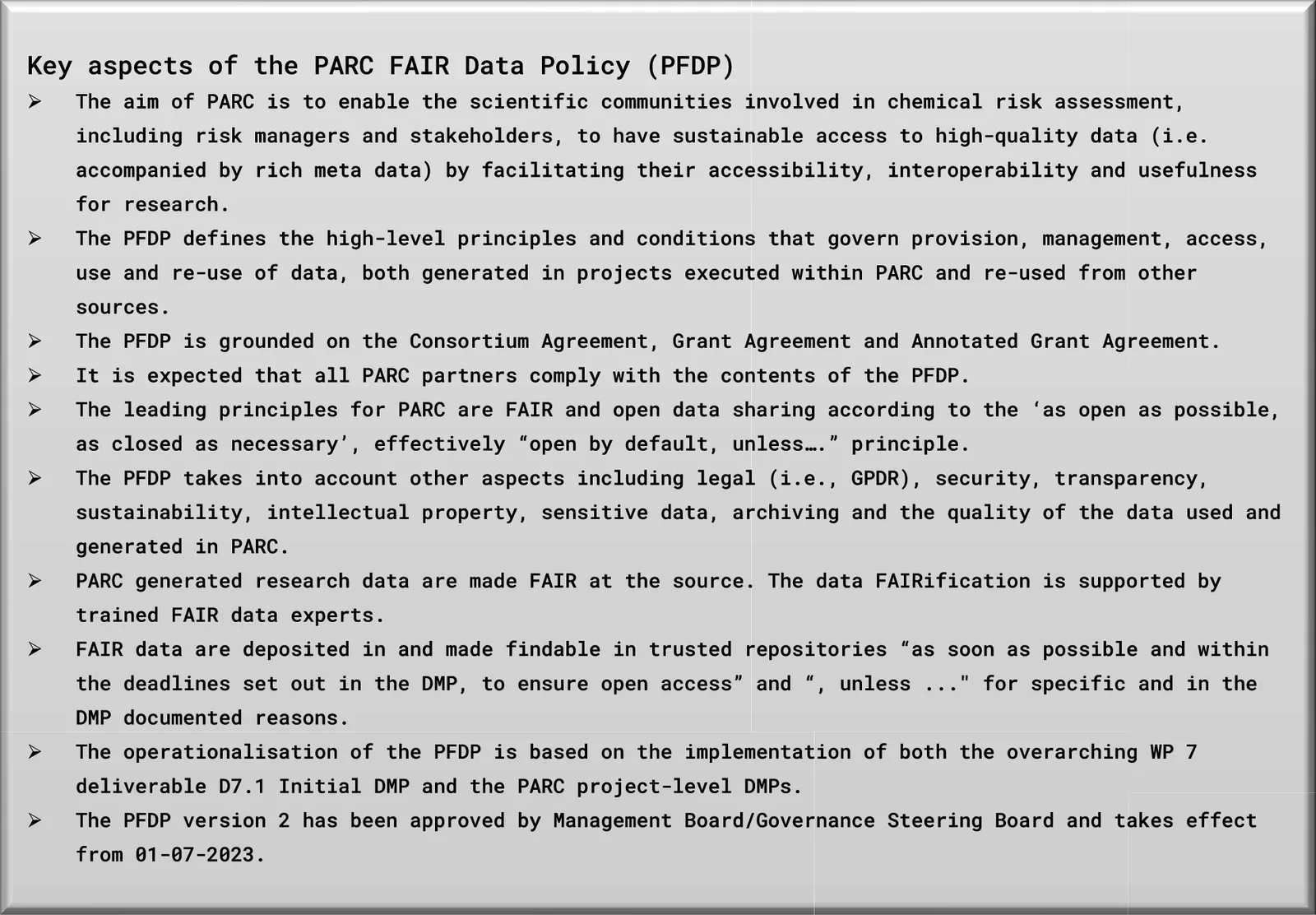
Overview of the content of the PFDP. For full access to the PFDP, see [11, 29]

Interestingly, while several topics were already identified during the expert session, the stakeholder document screening approach yielded new relevant topics (compare Supplementary Information Table S1 with Table 1), such as “applicable law and regulations”, “transparency and openness”, “intellectual property”, “data valuation”, and “sensitive data”. The taxonomy, together with the expert derived topics, served the creation of the first version of the PFDP. The following topics were included, as PFDP document headings: Aim and scope of the PARC FAIR Data Policy; Applicable laws and regulations; Data governance; Data management; Data security; Repositories; Transparency and openness; FAIR, including PARC’s Ambition in relation to FAIR data; Intellectual property; Sensitive data; Archiving. The writing of the draft PFDP was completed by the core group of 5 + 5 experts and the PFDP was distributed within PARC. The key aspects of the PFDP are shown in Fig. 5. The full text of the PFDP is available from Zenodo [29]. The metadata of the PFDP is described in more detail at FAIRsharing.org [11].

To build consensus and foster support PARC-wide for the PFDP already during its development, PARC members (Supplementary Information Table S3) were invited to provide input online and attend up to 3 from a set of repeated discussion sessions (6 in total, 2 per topic). Three individuals provided online input via Microsoft Forms. From the 332 individuals invited, 154 unique individuals were present in any of the 6 sessions (46.3% of invited individuals, ~ 9.9% of the PARC community) with some individuals attending multiple sessions. From these sessions, the following observations were made, which were considered to improve the PFDP.

With respect to the "Aim and scope of the PARC FAIR Data Policy”, a more descriptive definition of “data “ as suggested by Codata [4] was adopted (see PFDP [29]: page 12). It was discussed that assuring data quality is the responsibility of the researcher and compliance with agreements of (quality) standards should be described in project Data Management Plans (DMPs) (PFDP [29]: page 16). Although FAIRification can contribute to data quality, it was understood that this is not equal to data quality. In relation to repositories, a suggestion was made to use the IPCHEM portal [10] for chemical monitoring data. Concerning transparency and openness, it was concluded that data, should be “as open as possible, as closed as necessary”. In relation to this, legal boundaries (or, conversely, the absence of legal grounds), privacy issues—whether or not related to consent—and intellectual property rights may lead to situations where data disclosure is either impossible or only possible under specific restrictions (such as anonymization, subscription fees, etc.). Here, also the issue of a possible embargo for data release was brought up. It was raised (PFDP [29]: page 22) that: “PARC will strive for a minimum level of FAIRness for all PARC data and metadata, whether it is Open or Closed…”. Effectively this means that at least the project level metadata, and ideally the underlying data, should be FAIR and open and FAIRification is the responsibility of all researchers within PARC (page 9). On sensitive data, (informed) consent was mentioned, which is potentially more restrictive compared to other aspects in the GDPR. Informed consent is relevant, in particular in cases where study participants may express their wish to withdraw informed consent after data has been collected and processed. In relation to this, the importance of a data controller safeguarding the removal of [source] information was brought up (PFDP [29]: page 24). Finally, related to sensitive data, issues on data minimization (only give access on a need-to-know basis), anonymisation and pseudonymisation of data were brought into the discussion.

### Enhancement of the original topics using LLMs

To test the value of LLM for the discovery of novel topics, the terms (Table 1) were used as input into LLMs. GPT yielded initially a list of 49 terms from which, after de-duplication and exclusion of meaningless ‘noise’ terms, 40 terms were considered relevant (Supplementary Information Table S5). The novelty of these terms, in comparison to terms already proposed in the expert session and taxonomy, was further evaluated. Not surprisingly, some of these terms were identical or similar to the taxonomy. However, quite some terms, e.g., “vulnerability assessment”, “data anonymization”, “data breach”, “data encryption”, were indeed novel compared to the outcome of the expert panel and stakeholder document screening. Some of these terms were also raised during the preceding PARC consultation sessions (step 5, Fig. 3), prior to this exploration of the LLM approaches, and already included into the first version of the PFDP. 26 additional terms were finally considered to be novel, compared to the expert and taxonomy terms.

The outcome of using BERT combined with the stakeholder documents is shown in Supplementary Information Table S6. From the top 150 noun phrases obtained, 42 relevant terms were ultimately retained. Four terms (“data analytics”, “data integration”, “data sharing” and “data storage”) were also retrieved with GPT (Supplementary Information Table S5). Out of the 42 terms, 22 terms were identified as novel. Examples of some possible novel terms include “data coordination groups” and "data sharing agreement”.

### Frequency analysis within stakeholder documents of terms derived from the hierarchy of terms (taxonomy) and GPT and BERT LLM modelling

Supplementary Information Table S7 (Excel file) shows the outcome of analysing the frequency of the 135 terms within the 37 stakeholder documents. In column B-BF (orange), the 57 expert/taxonomy NTs are shown. In column BG-CT (purple) and column CU-EF (blue), terms from GPT and BERT are shown respectively. Concerning GPT, some of these terms were indeed mentioned within the 37 stakeholder documents, suggesting the discovery of possible relevant additional terms using this approach. Since BERT was used in the beginning to find terms similar to the 57 NTs within the stakeholder documents, it is evident that all the BERT terms appeared in at least one or more stakeholder documents. Together, this indicates that LLM assisted automated approaches of term retrieval are capable of identifying potential topics, within stakeholder documents spanning research, chemical RA and policy making, and (FAIR) data sciences.

Based upon the frequency analysis, stakeholder documents were clustered. As indicated (see Methods section) an initial PCA cluster analysis (step 13b, Fig. 4) performed on the outcome of the frequency analysis did not yield a satisfactory result, as the first three PCs only explained ~ 25% of the variation. Therefore, to aim for a more condensed cluster analysis, the 135 terms were first subjected to a FAIR data oriented grouping into “FAIR categories” using GPT (step 14, Fig. 4), see Supplementary Information Table S4. This additional step yielded 8 overarching “FAIR categories” named: “Data Governance and Policies”; “Data Management”; “Data Protection and Security”; “Data Sharing and Collaboration”; “Ethics and Fairness”; “Information Needs and Assessment”; “Policy and Governance”; “Technical Aspects”.

Without discussing these “FAIR categories” in detail, one interesting observation is that GPT apparently brings "Ethics and Fairness” together into one “FAIR category”, possibly as a result of the original meaning of the word ‘fair’ [2].

For each of the 8 GPT derived “FAIR categories”, we first counted how many times each of the individual underlying taxonomy and/or LLM derived terms within these FAIR categories occurred in the document (as was already done before for the initial frequency analysis based upon the individual terms only). Next, we then calculated the summation of these counts, that is we calculated the sum of the number of times all terms within a particular FAIR category were mentioned in the document. We then normalized these summations by dividing them by the total number of terms, out of all 135 terms, found in each document. The resulting normalized frequencies per overarching “FAIR Category” were subjected to PCA and K-means clustering. For clarity, these analyses were not based upon the frequency of the 8 GPT derived “FAIR categories” themselves, that is, as words/phrases counted within the text of the different stakeholder documents. Further, as stated earlier, the clustering was performed as explorative analysis only to assess the feasibility of identifying similarities between different stakeholder documents, and thus potentially differences between stakeholders as to their understanding of/emphasis on (FAIR) data for chemical risk assessment.

When using the outcome of this frequency analysis as input into PCA, a satisfactory model was generated as over 50% (19.49% (PC1) + 18.36% (PC2) + 14.97% (PC3) = 52.82%) of the variation in the data could be explained by the first three PCs (see Supplementary Information Figures S1-S2 and accompanying text). The outcome of PCA with regard to showing differences between stakeholder documents is largely identical to that from the K-means clustering described below.

**Fig. 6:**
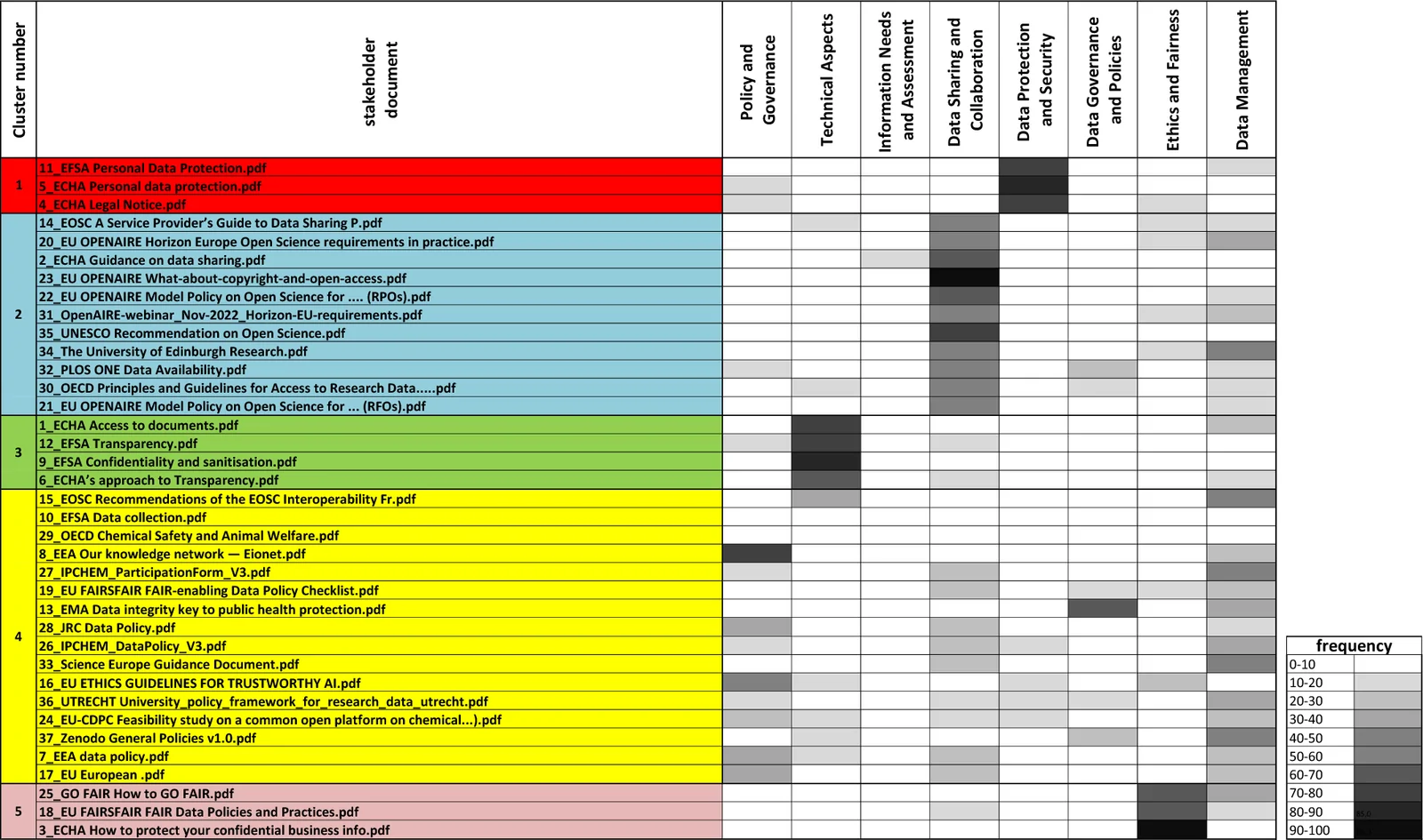
K-means clustering analysis performed on the normalized summation of the initial frequencies of the 135 terms (Supplementary Information Table S7 (Excel file)) under the 8 GPT derived “FAIR categories” (see Supplementary Information Table S4). These 8 “FAIR categories” are indicated as column headers. The different k-means clusters are indicated by different colors and a “cluster number”. The numerical values after summation and normalization of the term frequencies per overarching “FAIR category”, are shown and visualized using a grey scale (explained in the bottom right of the Figure). The stakeholder documents are indicated in the left column. The original documents can be found in “Supplementary Information Stakeholder Documents.pdf”

An overview of the outcome of K-means clustering is provided in Fig. 6. Cluster 1 (red) consists of three documents from EU agencies (ECHA and EFSA), in which in particular terms assigned to the GPT derived “FAIR category” named “Data protection and security” were frequently found. Cluster 2 (blue) consists of documents mainly from stakeholders/projects related to open science, open access, and/or research from public funding, such as EOSC, EU OPENAIRE, academic data management policies, and publishers (e.g. PLOS ONE). Within this cluster, primarily terms grouped under the “FAIR category” “Data Sharing and Collaboration” were frequently found, together with terms grouped under “Data Management”. Cluster 3 (green) consisted of four documents obtained from EU agencies (ECHA and EFSA) dealing with transparency, in which terms under the “FAIR category” “Technical Aspects” were grouped. Cluster 4 (yellow) consisted of documents obtained from a wide variety of stakeholders, including agencies involved in RA and policy making (EEA, EFSA), EU, OECD, EOSC etc. In this cluster, primarily terms under the “FAIR category” “data management” and to lesser extent terms under “data sharing and collaboration” contributed. Cluster 5 (pink) consisted of 3 documents from initiatives/organisations, involved in FAIR data and from ECHA. This cluster was characterized by a higher frequency of terms belonging under the “FAIR category” “Ethics and Fairness”.

Together, the summed and normalized frequencies of the 135 terms under the 8 GPT-derived “FAIR Categories” were subjected to K-means clustering and PCA (Supplementary Information Figure S1 and Figure S2). Using both methods, a separation into 4 to 5 main clusters and similarity between methods was observed in terms of the “FAIR categories” contributing to this separation.

## Discussion

Data sciences are becoming increasingly important to develop NGRA methods, helping to establish the “Chemicals Strategy for Sustainability Towards a Toxic-Free Environment” under Europe’s Green Deal. Novel research methods generate large amounts of data that need to be integrated with, and compared to, conventional data, to define their added value in NGRA and enable regulation to keep pace with chemicals innovation. PARC is at the heart of the research efforts needed to facilitate this transition, innovating in chemical RA for regulatory purposes. To foster the adoption of FAIR principles for data generated within PARC in the context of developing NGRA methods, a FAIR data policy (PFDP) was developed [11, 29] to guide all partners in their research data management activities. The development of the PFDP was based upon a unique combination of leveraging expert knowledge, stakeholder document analysis, PARC consortium wide discussions, and iterative cycles of writing by experts. An overview of the main topics of the PFDP is provided in Fig. 5.

While developing the PFDP, it became evident that incorporating the FAIR principles [31] alone was insufficient in the context of developing NGRA. Additional relevant topics needed to be incorporated. Examples of these needs included statements on “applicable laws and regulations”, as RA requires compliance with chemical legislation under e.g. REACH, but also with GDPR in datasets, where human subjects are involved. Further, the inclusion of additional information on data governance, data management and data security was deemed relevant. Examples are the description of specific roles in data management activities in PARC including data champions and data liaisons, who are guiding the FAIR annotation of PARC projects (see PFDP [29]: page 28).

Other examples were the need to include paragraphs on possible “Intellectual property” generated in PARC, and “Sensitive data” as a category of data besides data falling under the GPDR, involving possible commercially sensitive data. e.g. data from corporate risk assessment dossiers.

The discussions held during the expert session, together with screening of the stakeholder documents and PARC wide consultation sessions, proved to be a rigorous approach to successfully identify and include these additional topics. The latter were also useful to obtain commitment to, and understanding of, the PFDP by the broader PARC consortium.

Whereas the PFDP covers data generated by the totality of different scientific disciplines involved in innovating RA, previous studies considered or implemented FAIR principles for subdomains of RA. For nanosafety, Jeliazkova et al. [15] identified among others the need for persistent identifiers (PIDs), provision of rich metadata, indexed databases, and FAIR metadata schemas, which aligns with the PFDP which also requires the provision of PIDs and rich metadata. Ammar et al. [1] developed a computational workflow to assess the FAIRness of data deposited in life sciences databases and suggested the need for a much richer collection of FAIR metrics that reflect the domain-relevant community standards. In the context of human biomonitoring research, Zare Jeddi et al. [33] proposed developing a FAIR environment and health registry for epidemiological data to facilitate the reuse of human data. Ghosh et al. [12] described the development of data management concepts, FAIR-enabling tools, and secure data platforms for the EXIMIOUS exposome project, which generates heterogeneous biological and exposure data to study exposure-mediated immune effects and aligned project activities with the FAIR principles [31]. Furthermore, they developed a “DMP check” for assisting in the FAIRification of other projects and considered compliance with EU legal and ethical frameworks. Pineda-Pampliega [26] addressed the extent to which the EFSA OpenFoodTox and PlanetTracker’s Seafood databases aligned with the FAIR data principles. They proposed suggestions for accessibility, interoperability and reusability of data, for example via GitHub or Zenodo, in line with the PFDP [29] (as described on page 18”…data should be in a domain specific EU repository. Preference should be given to repositories …. (like e.g., Zenodo….””). In future, some of these efforts may also align with the objectives of the European Health Data Space (EHDS), particularly the development of the HealthData@EU infrastructure.

Heacock et al. [14] described how within the USA NIEHS Superfund Research Projects, data sciences can help to link exposures to health effects. They highlighted, similar to the PFDP, the importance of FAIRness. Issues encountered included the need for: research planning and collection of data; standards and ontologies; metadata annotations; infrastructure building; enabling artificial intelligence (AI) and machine learning (ML); and data protection needs. Further, the authors plea for building a collaborative research community of FAIR practice, is envisioned within PARC in a similar manner. Together, the findings from these studies are well in line with the independent experience we provide here.

The process of creating the PFDP for PARC has been quite intense and lasted over a year (~ October 2022 – October 2023). PARC is a long research initiative, running from 2022 to 2029, with the necessity to update the PFDP periodically to ensure it remains relevant and innovative. Therefore, as explorative analysis, we considered the possibility of identifying additional new topics of possible relevance by using LLM methods. Using GPT [23], we observed terms that were similar or identical to the taxonomy of terms retrieved via the expert session and screening of stakeholder documents. Examples of concepts identified via the LLMs are: “data sharing”; “data processing”; “data sharing guidelines”; “metadata”; “standardization”. This illustrates that LLMs are capable of confirming expert and stakeholder derived information. Moreover, some terms, e.g., “data anonymization” and “data breach”, were novel compared to the taxonomy. A similar observation was made using BERT [5]], in which, compared to the taxonomy, 22 novel terms were discovered (Supplementary Information Table S6). Obviously, there is some bias as to the expert judgement of the novelty of LLM derived terms. However, terms were found which, in hindsight and only following the initial creation of the taxonomy, proved to be relevant for the writing of the PFDP. Therefore, the use of LLM models can indeed assist in the retrieval of relevant novel terms/topics, helpful for future improvement and completion of PFDP updates.

In order to assess the diversity of documents obtained from different stakeholders used as the basis for the PFDP, we determined the frequency of occurrence of the final 135 terms (from the expert session, stakeholder documents and LLM). Condensing these further into 8 “FAIR categories” using GPT, enabled the successful clustering of stakeholder documents. Generally, the outcome of the K-means clustering and PCA (Supplementary Information Figure S1 and Figure S2) was similar. A first cluster generated by K-means clustering (Fig. 6, cluster 1 (red)) contained three documents from EU decentralized agencies (ECHA and EFSA), in which terms fitting to the GPT-derived “FAIR category” of “data protection and security”, were particularly enriched. Looking globally at these documents, a conclusion could be that for ECHA and EFSA, the aspect of data protection and security is important, for example in light of data obtained from humans.

Another document cluster (cluster 2 (blue), to which terms belonging to the GPT-derived “FAIR categories” of “Data Sharing and Collaboration” and “Data Management” contributed, was from stakeholders involved in open science or publicly funded research. Not surprisingly, for these stakeholders the collaboration on and sharing and management of data are relevant in order to foster open science. Cluster 3 (green) consisted of 4 documents obtained from ECHA and EFSA, dealing with transparency (among other topics). These documents displayed a relatively high abundance of terms assigned to the GPT-derived “FAIR category” “Technical Aspects”. Possibly, technical aspects related to data are relevant here as a means to promote the transparency of the information used for RA. Cluster 4 (yellow) consisted of documents obtained from a wide variety of stakeholders, (EEA, EFSA, EU, OECD, EOSC etc.). Here, overrepresented terms where part of the “FAIR categories” of “data management” and “policy and governance”. An observation might be that for both regulatory applications, as well as open science (EOSC), the issues of “data management” and (adhering to) “policy and governance” structures are relevant. Cluster 5 (pink), consisted of 3 documents, from ECHA and from FAIR data initiatives. This cluster was characterized by the enrichment of terms that corresponded to the “FAIR category” “Ethics and Fairness” (Fig. 6), which may be explained by the fact that the two documents from FAIR initiatives contained terms such as “FAIR” and “fair data principles” (see Supplementary Information Table S7). Together, based upon the differential clustering of the frequencies of terms, similarities and differences between stakeholders’ data management-related documents were observed. Organisations/initiatives involved in open science and publicly funded research could be largely distinguished from those involved in FAIR data, from those involved in the evaluation of the potential health risks associated with chemicals. In the future, this approach can be further developed to identify trends across different data domains relevant to RA. Examples of these could be the embracing of the FAIR principles in traditionally more “closed” regulatory risk assessment data domains.

Regulatory risk assessment (e.g., chemical safety dossiers) involves, understandably, restricted or proprietary data environments (e.g., IUCLID for ECHA submissions). The EU Common Data Platform on Chemicals (CDPC) [7] aims to establishes rules to enable users to discover data, ensure that chemicals-related information is interoperable and accessible, and define rights for access to information as well as for authorities to use data within the platform. Enhancing FAIRness in these data domains, and identifying this via term frequency analysis of FAIR categories as proposed here, can help to identify future FAIR data-driven possibilities to improve risk assessment. Examples of how such analysis could generate wider benefits include: promoting greater transparency in how decisions are made by improving discoverability and accessibility of underlying data; encouraging the convergence toward shared vocabularies, ontologies, and metadata standards between regulatory and research data, such as that generated in PARC. Other examples include the development of more holistic risk assessments, integrating multiple evidence streams that are currently fragmented; promoting faster knowledge transfer between regulatory, academic, and industrial domains; and raising opportunities for AI/ML applications, which depend on well-structured, interoperable data. Together with the anticipated increase in FAIRness of NGRA data within PARC, this has potential to speed up the data cycling between predictive and mechanistic models to improve RA (Fig. 1).

## Conclusions

In conclusion, a taxonomy of terms and from this a FAIR data policy (PFDP) for (next generation) risk assessment data was developed. The policy contributes to the ultimate reuse of environmental and human health research data for research purposes, aiming also to foster the adoption of research data in regulatory applications, bringing together different stakeholders interests. A unique methodological approach was followed, combining expert knowledge, the exploration of stakeholder documents and consultation rounds within the PARC project.

Relevant aspects identified were data governance and management, including security, quality and general FAIR data principles. Further, the FAIR ambition of PARC was included, outlining a minimum level of FAIRness for all PARC data and metadata. In addition, aspects specifically relevant for RA, such as GDPR, sensitive data, transparency, openness, and intellectual property and data valuation were identified.

The use of LLM and data analyses was successfully used to identify new topics relevant for the PFDP, which opens up the potential future exploration of trends amongst different stakeholders involved in RA, with respect to adopting FAIR data principles in research and/or regulatory data. The PFDP is adopted specifically within PARC but can serve as a guidance document for other environmental and health RA initiatives as well.

## Supplementary Information

SupplementaryMaterial 1

SupplementaryMaterial 2

SupplementaryMaterial 3

SupplementaryMaterial 4

SupplementaryMaterial 5

SupplementaryMaterial 6

SupplementaryMaterial 7

SupplementaryMaterial 8

SupplementaryMaterial 9

## Data Availability

All data generated/analysed during this study are included in this published article, and its Supplementary Information Figures (S1-S2) and Tables (S1-S7). The underlying/supporting PARC FAIR Data Policy to this publication is available at Zenodo. https://doi.org/10.5281/zenodo.15005810The PARC FAIR Data Policy metadata is described in detail under FAIRsharing.org: https://doi.org/10.25504/FAIRsharing.e700be

## Abbreviations

AGA: Annotated grant agreement
AI: Artificial intelligence
AOP: Adverse outcome pathway
BERT: Google’s BERT base model
BT(s): Broader term(s)
CA: Consortium agreement
CAS*: Chemical abstracts service
CC*: Creative commons
COPCSD: Common open platform on chemical safety data
DEPB*: Data protection and ethics board
DG ENV*: Directorate-general for environment
DMP: Data management plan
DMP*: Data model
DPIA*: Data protection impact assessment
EC*: European commission
ECHA: European chemicals agency
EEA: The European environment agency
EFSA: European Food Safety Authority
EHDS: European Health Data Space
EMA: The European Medicines Agency
EOSC: European open science cloud
EU: European Union
FAIR: Findable, Accessible, Interoperable and Reusable
GA: PARC Grant Agreement
GB*: Governing Board
GDPR: General Data Protection Regulation
GLP*: Good laboratory practice
GPT: OpenAI’s GPT model (version 3.5-turbo)
GSB*: Grant signatory board
HTS: High-throughput Screening
IP*: Intellectual property
IPCHEM*: Information platform for chemical monitoring
IPR*: Intellectual property rights
JRC*: Joint Research Center
LLMs: Large Language Models
MB*: Management Board
MIE: Molecular Initiative Event (start of an AOP)
ML: Machine learning
NAMs: New-approach methodologies
NGRA: Next-Generation Risk Assessment
NH*: National Hub
NIEHS: National Institute of Environmental Health Sciences
NLP: Natural language processing
NT(s): Narrower term(s)
OA*: Open Access
OECD: Organisation for Economic Co-operation and Development
PARC: Partnership for the assessment of risk from chemicals
PCA: Principal component analysis
PC(s): Principal component(s)
PFDP: PARC FAIR data policy
PIDs: Persistent identifiers
PLOS*: Public Library of Science
qAOP: Quantitative Adverse Outcome Pathway
QSAR*: Quantitative structure activity relationship
RA: Risk assessment
RDM*: Research data management
RPO*: Research Performing Organisations
TFEU*: Treaty on the Functioning of the European Union
TL*: Task leader
UK: United Kingdom
USA: United States
WP*: Work package
