# Supplementary material for: Developing a FAIR data policy for chemical risk assessment and exploring future topics and stakeholder interests with large language models and data sciences
Source: Environ Sci Eur. 2026 Jul 26;38(1):205. doi: 10.1186/s12302-026-01472-y (PMC13687255; doi:10.1186/s12302-026-01472-y)
Supplement: Supplementary file 1 [file 12302_2026_1472_MOESM1_ESM.pdf]

Accompanying text to Supplementary Information Figure S1 and Supplementary Information Figure S2.

Considering the outcome of the PCA analysis performed on the summated and normalized frequencies of the terms under the GPT-derived "FAIR categories", the first three PCs explained over half of all the variance in the data (19.49% (PC1) + 18.36% (PC2) + 14.97 % (PC3) = 52.82%). This is a significantly improvement over the PCA performed on the 135 frequencies (<25%) allowing for a more meaningful interpretation of the cluster models. Supplementary Information Figure S1 shows the outcome of the PCA performed on results of the frequency analysis of the 135 terms divided over 8 "FAIR categories". Each dot represents a PCA score for each individual stakeholder document. In Supplementary Information Figure S1A, the scores of PC2 are plotted against the scores of PC1. Similar visualizations for PC3 versus PC1 and PC3 versus PC2 are shown in Supplementary Information Figure S1B and S1C, respectively. The coloring of each of the respective scores is based upon the K-means cluster in which the stakeholder document was clustered. In Supplementary Information Figure S2, the PCA loadings are represented for PC1, PC2, and PC3. These are the GPT-derived "FAIR categories" contributing most to the separation of the scores (stakeholder documents). As is evident from Supplementary Figure 1, the PCA scores, derived from the stakeholder documents that ended up into the blue and yellow K-means clusters, are mainly separated on PC1 from the scores corresponding to the green and red K-means clusters (Supplementary Information Figure S1A and S1B, x-axis). If one considers this together with the corresponding loadings (Supplementary Information Figure S2A), positive loadings (that is GPT-derived "FAIR categories") are "Data protection and security" and "Technical Aspects", in the direction of the scores mainly labeled with green and red colours. This is in line with the results of the K-means clustering (Figure 6) which was mainly driven by terms belonging to the "FAIR category" named
"Data protection and security" (highest frequency in cluster 1 (red)) and "Technical Aspects" (highest frequency in cluster 2 (green)). Negative loadings are "Data Sharing and Collaboration", "Data Governance and Policies" and "Data management", in the direction of the scores labeled with blue and yellow colours, also in line with the results from K-means analysis (blue cluster: highest frequency of "Data Sharing and Collaboration", followed by "Data Management"; yellow cluster highest frequency of "data Management"; followed by "Data sharing and collaboration". On PC2 (Supplementary Information Figure S1A, y-axis, Supplementary Information Figure S1C, x-axis), the PCA scores derived from stakeholder documents that clustered within the K-means cluster 2, (blue), are, in general, separated from the scores, obtained from all other documents belonging to all other K-means clusters. The loadings (positive scores) (Supplementary Information Figure 2B) contributing to this separation are "Information needs and assessment", "Data sharing and collaboration", and "Ethics and Fairness", which generally fits some of the observations obtained after K-means clustering (e.g. high frequency on "data sharing and collaboration"). On PC3 (Supplementary Information Figure S1B and S1C, y-axis) , the scores derived from documents belonging to the K-means cluster 3 (green) are separated from the other clusters. The loading ("FAIR Categories") mostly contributing to this (Supplementary Information Figure S2C) is "Technical aspects", in line with the outcome of K-means (Figure 6).

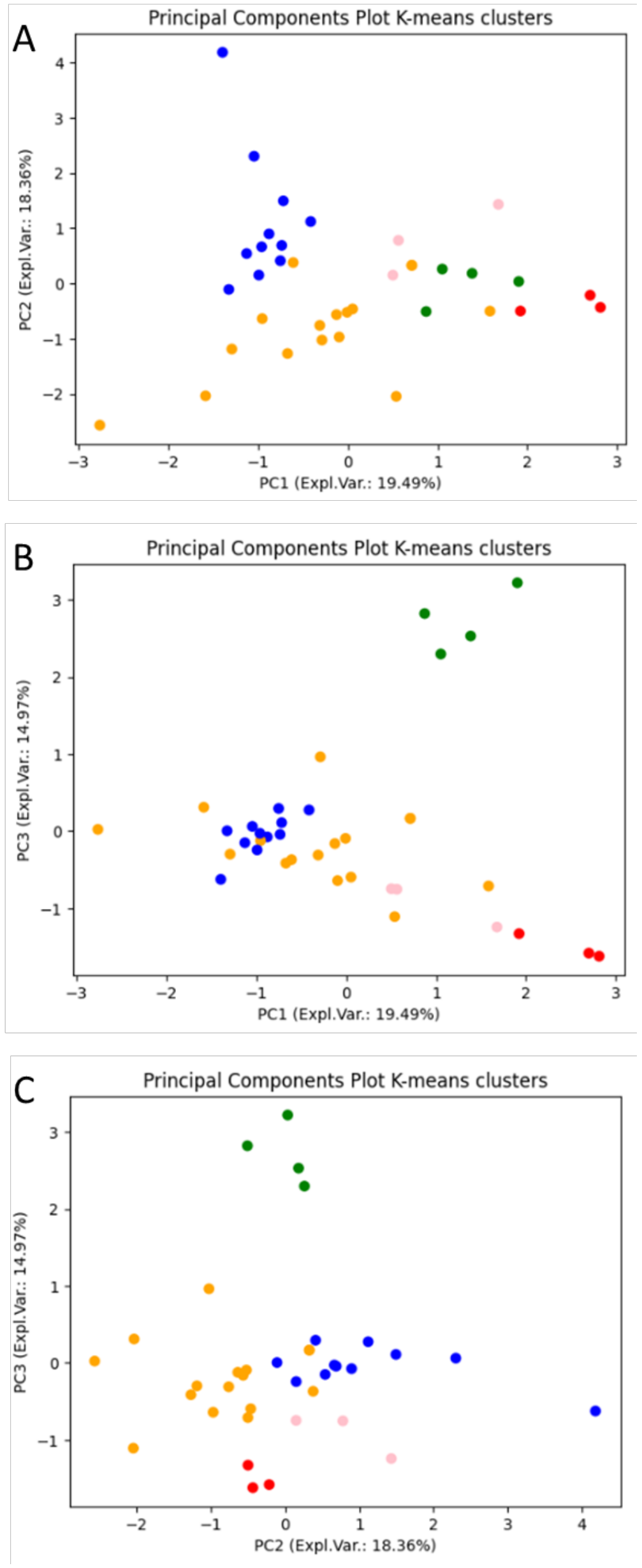

Supplementary Information Figure S1. PCA performed on the outcome of frequency analysis, performed within the 37 stakeholder documents, of 135 terms categorized into 8 FAIR data oriented clusters. Each dot represents the PCA score belonging to a stakeholder document. The dot colors represent the clusters observed with K-means (Figure 6) performed on the frequency analysis of the 8 terms. Supplementary Figure S1A: PC2 vs PC1; S1B:PC3 vs PC1; S1C: PC3 vs PC2.

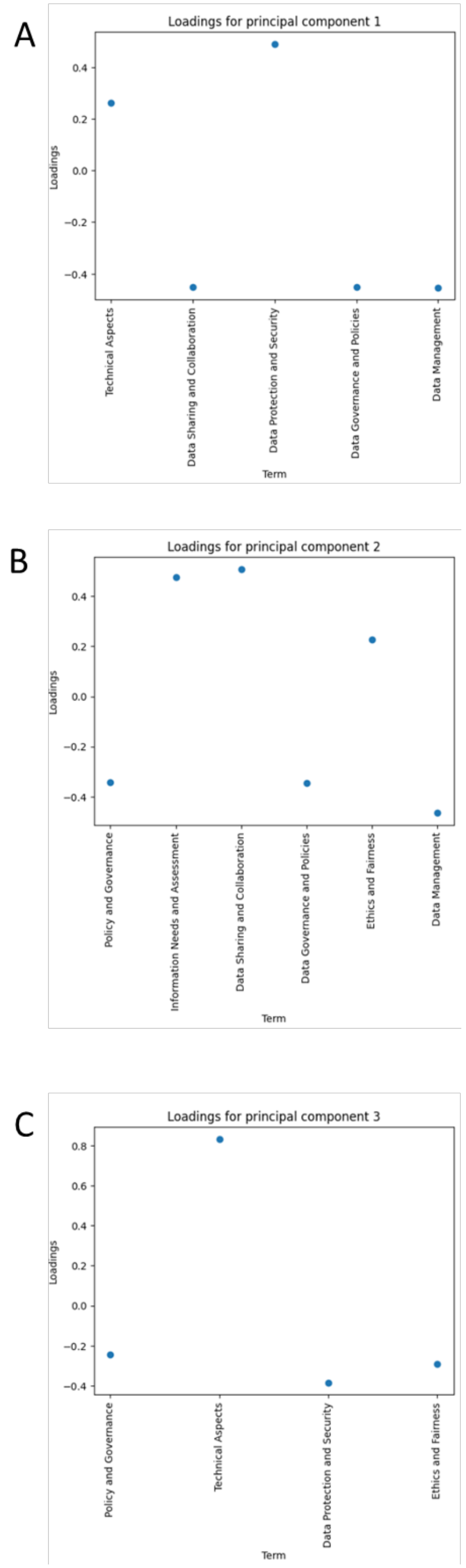

Supplementary Information Figure S2. PCA performed on the outcome of frequency analysis of 135 terms categorized under 8 GPT-derived “FAIR categories”, within the 37 stakeholder documents. Each dot represents the loading (term) on the respective PC. Supplementary Figure 2A: PC1; Supplementary Figure 2B: PC2; Supplementary Figure 2C: PC3. Comparing these plots with those in Supplementary Information Figure S1 enables evaluation, per PC, of the extent to which the loadings (summed and normalized frequencies of subsets of the 135 terms under the “FAIR categories”) contribute to separation of the scores within the PCA clusters. Note that higher (positive or negative) loadings imply a greater contribution to the separation, while the sign of the loading indicates if the variables are positively or negatively correlated.
