# Supplementary material for: Developing a FAIR data policy for chemical risk assessment and exploring future topics and stakeholder interests with large language models and data sciences
Source: Environ Sci Eur. 2026 Jul 26;38(1):205. doi: 10.1186/s12302-026-01472-y (PMC13687255; doi:10.1186/s12302-026-01472-y)
Supplement: Supplementary file 3 [file 12302_2026_1472_MOESM3_ESM.docx]

| **Supplementary Information Table S1. Possible relevant topics for inclusion into the PFDP, as identified during a two- day expert^1^ meeting held in Brno, Czech Republic.** | |
| --- | --- |
| **Snippets from original notes made at the Expert Meeting** | **Final topics identified at the Expert Meeting** |
| metadata, identifiers are made FAIR | FAIR metadata |
|  | FAIR identifiers |
| PARC recommendation on fair levels for: | level of PARC level of fairness |
| raw data (depends on type data eg omics), | raw data |
| processed data, | processed data |
| summary data | summary data |
| data need an appropriate license | data need an appropriate license |
| minimum fair criteria for summary data | minimum fair criteria for summary data |
| provenance metadata | provenance metadata |
| share and reuse for other (EU) projects | share and reuse for other (EU) projects |
| formats: metadata in RDF, data dictionary, data csv/excel files | data formats |
| for the PFDP refers functional requirements on this and refers to community standards. | community standards |
| the PFDP is dynamic as we learn from the pilot projects | version and updates of the PFDP |
| other than data digital objects: protocols, semantic artifacts in a parc repository (ideally fair) eg PARCopedia (is it FAIR?) | PARC repository (e.g. parcopedia (is it fair?) ) for, other than data: protocols, semantic artefacts? |
| [PARC] WP8 task: what about the tools, models and what about making them FAIR?^2^ | scope of the PFDP (e.g. software) |
| ^1^These 50 experts had backgrounds in sciences and associated data disciplines relevant to PARC, specifically: agricultural engineering; applied AI; bioengineering; biomedical semantics; cell biology; chemistry; chemometrics; computational and applied mathematics; computational biology; computational chemistry; computational toxicology; data driven knowledge discovery; data engineering; data sciences; databasing; ecological chemistry; ecotoxicology; environmental chemistry; environmental health risk analysis; environmental nanoscience; exposure sciences; FAIR data; geology; geosciences; human biomonitoring; hydrogeology; machine learning; pharmaceutical bioinformatics; physiologically based pharmacodynamic modelling; remote sensing; risk assessment; software and data architecture; statistics; text mining; toxicology.  ^2^Within PARC “WP8: Concepts and toolboxes”, the FAIR principles, data and tools have been further discussed specifically for operationalisation of Safe and Sustainable by design, as described in [15]. For a more detailed general description of WP8 (and other WPs) as described in [17]. | |
