# Supplementary material for: Developing a FAIR data policy for chemical risk assessment and exploring future topics and stakeholder interests with large language models and data sciences
Source: Environ Sci Eur. 2026 Jul 26;38(1):205. doi: 10.1186/s12302-026-01472-y (PMC13687255; doi:10.1186/s12302-026-01472-y)
Supplement: Supplementary file 4 [file 12302_2026_1472_MOESM4_ESM.docx]

| **Supplementary Information Table S2. Overview of 37 stakeholder documents used to further develop the PFDP. The stakeholders were identified by 5 PARC scientists^1^ and documents from these were extracted from websites for which the original hyperlinks (column “hyperlink”) are provided. The PDF documents refer to the downloaded content (either the website or the underlying documentation) used as input into frequency analysis (step [13], Figure 3). These are also available in “Supplementary Information Stakeholder Documents.pdf”. The column “prioritized content” cites the text snippets extracted from these documents/websites, or describes, in case of further expert interpretation, the textual information screened.** | | | | | |
| --- | --- | --- | --- | --- | --- |
| **Number** | **Stakeholder type** | **Stakeholder** | **PDF documents, for each of the 37 documents available in PDF portfolio file “Supplementary Information Stakeholder Documents.pdf”** | **hyperlink** | **prioritized content** |
| 1 | EU Agency | ECHA | 1_ECHA Access to documents.pdf | [Access to documents](https://echa.europa.eu/about-us/the-way-we-work/procedures-and-policies/access-to-documents) | #transparancy (EU access to documents for citizen and resident of European Union (‘EU’) countries |
| 2 | EU Agency | ECHA | 2_ECHA Guidance on data sharing.pdf | [Data sharing](https://echa.europa.eu/documents/10162/2324906/guidance_on_data_sharing_en.pdf/545e4463-9e67-43f0-852f-35e70a8ead60) | #data “ancienity”, [“ancienity” was used initially to described the time past since data creation]: open after x number of years #data sharing practical guidance on sharing in relation to company costs. Promotion of data sharing to joint submissions and reduction of animal testing (incentive for data sharing) #data quality Klimisch |
| 3 | EU Agency | ECHA | 3_ECHA How to protect your confidential business info.pdf | [Practical example for the R2018 portal (europa.eu)](https://echa.europa.eu/documents/10162/2467020/example_how_to_protect_cbi_en.pdf) | #confidentiality, in particular a provision with guideline how to protect confidential business information (e.g. non-disclosure of company name in case of being a co-registrant in registration of a chemical for ECHA) |
| 4 | EU Agency | ECHA | 4_ECHA Legal Notice.pdf | <https://echa.europa.eu/legal-notice> | #use of data #legal constraints/protection of certain [Intellectual Property Rights] (IPR)^2^ (e.g. CAS numbers) from ECHA website. For purpose of scientific research text and data mining allowed// something on the use of cookies/duration of storation when visiting ECHA database website |
| 5 | EU Agency | ECHA | 5_ECHA Personal data protection.pdf | [Personal data protection - ECHA (europa.eu)](https://echa.europa.eu/about-us/the-way-we-work/personal-data-protection) | #GDPR: This policy also provides detailed guidance descriptions on the different aspects of personal data protection for different purposes (e.g. human resources, regulatory processes, administrative processes, communication processes). Be aware that GDPR can be at different levels! |
| 6 | EU Agency | ECHA | 6_ECHA’s approach to Transparency.pdf | <https://echa.europa.eu/documents/10162/17208/mb_61_2014_echa_transparency_en.pdf/068580f5-a523-4fb0-9fb8-90fd27c5277a?t=1419327138730> | #transparency: explain activities and processes; open decision making; making information available. important remark on balancing transparency against other core values (e.g. protection of IPR and confidential business information) |
| 7 | EU Agency | EEA | 7_EEA data policy.pdf | <https://www.eea.europa.eu/legal/eea-data-policy> |  |
| 8 | EU Agency | EEA | 8_EEA Our knowledge network — Eionet.pdf | <https://www.eea.europa.eu/about-us/countries-and-eionet> |  |
| 9 | EU Agency | EFSA | 9_EFSA Confidentiality and sanitisation.pdf | [Confidentiality and sanitisation \| EFSA (europa.eu)](https://www.efsa.europa.eu/en/applications/confidentiality-sanitisation) | # Copyright notice: Copyright © for the entire content of this website unless otherwise stated: European Food Safety Authority – EFSA. Re-use is authorised, provided that EFSA is acknowledged as the source of the material. For individual documents, the general principle of re-use outlined above may be subject to specific conditions as indicated in individual copyright notices contained therein. Users are advised to refer to these individual copyright notices. The EFSA logo is the exclusive property of the European Food Safety Authority – EFSA and is registered under Article 6ter of the Paris Convention for the protection of industrial property. Its use is prohibited without the prior written permission of EFSA. # Linking policy: EFSA encourages organisations and individuals to create links to its website. Linking should not: be used in a defamatory context; be displayed in a manner which suggests that EFSA endorses any commercial product or service, scientific or administrative output, or the information or text in the context of which it is published; confuse users of the other website as to the source of the content. EFSA should be fully acknowledged as a source or author of the relevant documents; e displayed alongside advertising. Some EFSA pages link to other websites maintained by external parties, over which EFSA has no control and for which EFSA is not responsible. Linking to another website should not be taken as an endorsement by EFSA. Users clicking and visiting another site are subject to the policies applicable to the new site. # Disclaimer: The European Food Safety Authority (EFSA) maintains this website to enhance public access to information about its activities and initiatives. Our goal is to keep this information timely and accurate. If errors are brought to our attention, we will try to correct them as soon as possible. Information on the EFSA website: reflects the information and state of knowledge available on the date it was adopted or endorsed; is not necessarily comprehensive, complete, accurate or up to date; is not to be interpreted as professional or legal advice (if you need specific advice, you should always consult a suitably qualified professional). |
| 10 | EU Agency | EFSA | 10_EFSA Data collection.pdf | [Data collection](https://www.efsa.europa.eu/nl/resources/data-collection) | # data upload form template: https://www.efsa.europa.eu/en/data/data-standardisation # Conversion of national values to EFSA catalogue terminology: SSD2 requires the mapping to EFSA coded terminologies and the FoodEx2 system requires a complex mapping to both base terms and facets to fully classify and describe the samples taken. EFSA now provides a catalogue browser application (EFSA, online-b) which links to the latest version of the catalogue and web services to enable better conversion. # Public access to document requests and Open Data. In recent years there have been many requests for access to datasets, largely under the Regulation on Public Access to Documents19. EFSA, in collaboration with Member States, has moved to an Open by Default approach for data in the Scientific Data Warehouse (29). However, this requires that sensitive information, e.g. personal data and commercially sensitive data, is protected. Therefore, the use of free text fields in ChemMon data submissions has been reduced and only those where the content is clearly specified remain. # making FAIR machine-actionable: All resources linked to this document (structural metadata, catalogues, business rules, schema definitions) will be published in the EFSA Knowledge Junction in a machine-readable format and human-readable where appropriate. |
| 11 | EU Agency | EFSA | 11_EFSA Personal Data Protection.pdf | [Personal Data Protection](https://www.efsa.europa.eu/en/personal-data-protection#:~:text=EFSA%20will%20process%20any%20personal,an%20Agency%20of%20the%20EU.) | # Respect for private and family life: Everyone has the right to respect for his or her private and family life, home and communications. (Article 7). # Protection of personal data: Everyone has the right to the protection of personal data concerning him or her. Such data must be processed fairly for specified purposes and on the basis of the consent of the person concerned or some other legitimate basis laid down by law. Everyone has the right of access to data which has been collected concerning him or her, and the right to have it rectified. Compliance with these rules shall be subject to control by an independent authority. (Article 8) The protection of natural persons in relation to the processing of personal data is a fundamental right. Article 8(1) of the Charter of Fundamental Rights of the European Union (the ‘Charter’) and Article 16(1) of the Treaty on the Functioning of the European Union (TFEU) provide that everyone has the right to the protection of personal data concerning him or her. This right is also guaranteed under Article 8 of the European Convention for the Protection of Human Rights and Fundamental Freedoms |
| 12 | EU Agency | EFSA | 12_EFSA Transparency.pdf | <https://www.efsa.europa.eu/en/about/transparency> | # Transparency: The regulation was introduced with the aim of increasing the transparency of risk assessment in the food chain. Citizens will have access to studies and information submitted by industry in the risk assessment process.  EFSA will be made aware of all commissioned studies to guarantee that companies applying for authorisations submit all relevant information. The Authority will also offer advice to applicants prior to the submission of dossiers. The European Commission may ask EFSA to commission additional studies and may carry out fact-finding missions to verify that laboratories/studies comply with quality standards. It is necessary, therefore, to ensure transparent, continuous and inclusive risk communication throughout the risk analysis, involving Union and national risk assessors and risk managers. Such risk communication should strengthen citizens' trust that the risk analysis is underpinned by the objective of ensuring a high level of protection of human health and consumers' interests. That risk communication should also be capable of contributing to a participatory and open dialogue between all interested parties in order to ensure that the prevalence of the public interest, and accuracy, comprehensiveness, transparency, consistency and accountability are taken into account in the risk analysis process. Information should be provided on how risk management decisions were reached and on the factors, other than the results of the risk assessment, which were considered by the risk managers, as well as how those factors were weighed up against each other. Transparency of the risk assessment process contributes to greater legitimacy of the Authority being acquired in the eyes of the consumers and general public in the pursuit of its mission, increases their confidence in its work and ensures that the Authority is more accountable to the Union citizens in a democratic system. It is therefore essential to strengthen the confidence of the general public and other interested parties in the risk analysis underpinning the relevant Union law, and in particular in the risk assessment, including the transparency thereof as well as the organisation, functioning and independence of the Authority. # Standard data formats: For the purposes of point (c) of Article 38(1) and in order to ensure the efficient processing of requests to the Authority for a scientific output, standard data formats shall be adopted in accordance with paragraph 2 of this Article to allow documents to be submitted, searched, copied and printed, while ensuring compliance with regulatory requirements set out in Union law. # Transparency and confidentiality: The Authority shall make public the application for authorisation, relevant supporting information and any supplementary information supplied by the applicant, as well as its scientific opinions, in accordance with Articles 38 to 39e of Regulation (EC) No 178/2002, which shall apply mutatis mutandis. |
| 13 | EU Agency | EMA | 13_EMA Data integrity key to public health protection.pdf | [Data integrity: key to public health protection \| European Medicines Agency (europa.eu)](https://www.ema.europa.eu/en/news/data-integrity-key-public-health-protection) |  |
| 14 | European Commission | EOSC | 14_EOSC A Service Provider’s Guide to Data Sharing P.pdf | [InfoCards_A4_June2020 v7.pdf (eosc-hub.eu)](https://www.eosc-hub.eu/sites/default/files/InfoCards_A4_June2020%20v7.pdf) | # Provenance: Information about provenance of scientific data is crucial to assess data integrity and authenticity.  # Policy accreditation: For providers of data and data services, external accreditation is becoming highly desirable, and sometimes essential, in building the necessary trust with important user communities, partner service providers, or both. Whether the right path for you is CoreTrustSeal, ISO27001, FitSM, or even ISO16363, formalising your policies on data sharing is a key first step, and of itself a great way to build trust with your existing user base. Formal policies will also help you create networks with your partners to support emerging research data codes of conduct across shared user communities.  # FAIR: All shareable data objects in the [European Open Science Cloud] (EOSC)-hub ecosystem should be FAIR Digital Objects.  # Licenses: Data objects in the EOSC-hub ecosystem should adopt licences from the Creative Commons 4.0 licence suite.  # Sensitive data: Within Medical and Health data services, a good domains practice is that of the Five Safes. EOSC-hub should adopt these Five Safes principles as guidance10 for the management and handling of sensitive data in the EOSC-hub ecosystem. |
| 15 | European Commission | EOSC | 15_EOSC Recommendations of the EOSC Interoperability Fr.pdf | <https://op.europa.eu/en/publication-detail/-/publication/d787ea54-6a87-11eb-aeb5-01aa75ed71a1/language-en> | # Interoperability: Our view on interoperability does not only consider data but also the many other research artefacts that may be used in the context of research activity, such as software code, scientific workflows, laboratory protocols, open hardware designs, etc. It also considers the need to make services and e-infrastructures as interoperable as possible. # Technical interoperability: Recommendations:  ● Open Specifications for EOSC Services. ● A common security and privacy framework (including Authorisation and Authentication Infrastructure). ● Easy-to-understand Service-Level Agreements for all EOSC resource providers. ● Easy access to data sources available in different formats. ● Coarse-grained and fine-grained dataset (and other research object) search tools.  ● A clear EOSC [Persistent Identifier] (PID) policy. # Semantic interoperability: Recommendations: ● Clear and precise, publicly-available definitions for all concepts, metadata and  data schemas.● Semantic artefacts preferably with open licenses. ● Associated documentation for semantic artefacts. ● Repositories of semantic artefacts, rules with a clear governance framework.● A minimum metadata model (and crosswalks) to ease discovery over existing federated research data and metadata. ● Extensibility options to allow for disciplinary metadata.● Clear protocols and building blocks for the federation/harvesting of semantic artefacts catalogues. Organisational interoperability: Recommendations: ● Interoperability-focused rules of participation recommendations. ● Usage recommendations of standardised data formats and/or vocabularies, and with their corresponding metadata.● A clear management of permanent organisation names and functions. Legal interoperability: Recommendations: ● Standardised human and machine-readable licenses, with a centralised source of knowledge and support on copyright and licenses. ● Permissive licenses for metadata (and preferably for data, whenever possible). And [Creative Commons, (CC)] CC0 preferred over CC BY 4.0.● Identification of different parts of a dataset with different licenses.● Clearly marked instances of expired or inexistent copyright, as well as for orphan data.● A clear list of EOSC-recommended licenses and their compatibility with Member States’ recommended licenses. ● Tracking of license evolution over time for datasets.● Harmonised policy and guidance to dealing with cases where patent filing or trade secrets may be compromised by disclosure.● GDPR-compliance for personal data. ● Additional restrictions on access and use of data only applied in cases of applicable legislation or legitimate reasons.● Harmonised terms of use across repositories● Alignment between Member States national legislations and EOSC. |
| 16 | [EU](https://ec.europa.eu/info/sites/default/files/summary-data-governance-data-policies_en.pdf) | [EU](https://ec.europa.eu/info/sites/default/files/summary-data-governance-data-policies_en.pdf) | 16_EU ETHICS GUIDELINES FOR TRUSTWORTHY AI.pdf | <https://ec.europa.eu/futurium/en/ai-alliance-consultation.1.html> | Privacy and data governance (p17): # Privacy and data protection. # Quality and integrity of data. : The quality of the data sets used is paramount to the performance of AI systems.  When data is gathered, it may contain socially constructed biases, inaccuracies, errors and mistakes. This needs to be addressed prior to training with any given data set. In addition, the integrity of the data must be ensured.  Feeding malicious data into an AI system may change its behaviour, particularly with self-learning systems.  Processes and data sets used must be tested and documented at each step such as planning, training, testing and  deployment. This should also apply to AI systems that were not developed in-house but acquired elsewhere. # Access to data. : In any given organisation that handles individuals’ data (whether someone is a user of the system or not), data protocols governing data access should be put in place. These protocols should outline who can access  data and under which circumstances. Only duly qualified personnel with the competence and need to access  individual’s data should be allowed to do so. # Traceability.: . The data sets and the processes that yield the AI system’s decision, including those of data gathering  and data labelling as well as the algorithms used, should be documented to the best possible standard to allow for  traceability and an increase in transparency.  # Explainability.: Explainability concerns the ability to explain both the technical processes of an AI system and the  related human decisions (e.g. application areas of a system). T  Communication.: AI systems should not represent themselves as humans to users; humans have the right to be  informed that they are interacting with an AI system. |
| 17 | [European Commission](https://ec.europa.eu/info/sites/default/files/summary-data-governance-data-policies_en.pdf) | [European Commission](https://ec.europa.eu/info/sites/default/files/summary-data-governance-data-policies_en.pdf) | 17_EU European .pdf | [https://commission.europa.eu/publications``/data-governance-and-data-policies-european-commission_en](https://commission.europa.eu/publications%60%60/data-governance-and-data-policies-european-commission_en) | # Governance versus policies: Data governance sets out a framework with clear roles, and the responsibilities and interdependencies of those roles. Data policies introduce common principles, guidance and working practices in the areas of data management, data interoperabilityand standards, and data quality. # Data assets: Data assets may be structured or unstructured, static or dynamic, raw or curated. Data assets are in digital format. # Implementation of data governance and policies: 1 – Definition & set up, 2 – (Pilot) implementation, 3 – Roll-out & operation # Guiding principles: 9 principles are introduced covering Embedding, Sustainability, Measurability, Accountability and responsibility, Transparency, Principle-driven, Community-wideness, Proportion, Comply-o-explain concepts |
| 18 | EU Project | EU FAIRSFAIR | 18_EU FAIRSFAIR FAIR Data Policies and Practices.pdf | <https://www.fairsfair.eu/fairsfair-open-consultation-fair-data-policies-and-practices> |  |
| 19 | EU Project | EU FAIRSFAIR | 19_EU FAIRSFAIR FAIR-enabling Data Policy Checklist.pdf | <https://zenodo.org/record/6225775#.YvJqBfhBz-g> | Scope of the policy.  # Definition of research data # Research software # Data sharing # Alignment with FAIR # Metadata sharing # [Data Management Plan] (DMP): Timing of DMP; Updating of DMP  # Data Protection # Research integrity  # Reference to standards # Repositories  # Data Availability Statement # Intellectual Property (IP)  # Licences # Data Citation  # Researcher Identifiers # Preservation |
| 20 | EU | OpenAIRE | 20_EU OPENAIRE Horizon Europe Open Science requirements in practice.pdf | <https://www.openaire.eu/horizon-europe-open-science-requirements-in-practice#:~:text=On%20June%2014%20OpenAIRE%20hosted,(OpenAIRE)%20and%20Pedro%20Pr%C3%ADncipe> | # tools / instruments to validate/re-use results and data # [Open Access] (OA) publishing and data availability statement  # trusted repositories for data  # licencing or other restrictions # order of applicability of: laws, contracts, funder requirements, policies, guidelines (what goes before what)  # spreadsheed data instructions |
| 21 | EU | OpenAIRE | 21_EU OPENAIRE Model Policy on Open Science for ... (RFOs).pdf | <https://www.openaire.eu/model-policy-on-open-science-for-research-funding-organisations> |  |
| 22 | EU | OpenAIRE | 22_EU OPENAIRE Model Policy on Open Science for .... (RPOs).pdf | <https://www.openaire.eu/model-policy-on-open-science-for-research-performing-organisations> | # Researchers responsibility: Researchers are responsible for Managing publications, data and educational resources in adherence with the principles and requirements expressed in this Policy.5# Researchers responsibility: Researchers are responsible for Ensuring that the principles governing the handling of data (in adherence with the present Policy and funders’ mandates) are included in a Data Management Plan (DMP).  # Researchers responsibility: Researchers are responsible for Compiling a DMP for every research activity they are coordinating. # Researchers responsibility: Researchers are responsible for Choosing the appropriate type of licensing for their research outputs.  # Open Access to Research Data: The [Name of RPO] ([Research Performing Organisations] (RPO)) Requires researchers to deposit the data needed to validate the results presented in scientific publications in a suitable repository such as [Name of Repository]. Data should be provided with persistent identifiers and must be linked with publications where possible. # Open Access to Research Data: The [Name of RPO] Requires that data and services are handled according to open and FAIR principles (i.e. Findable, Accessible, Interoperable and Re-usable). Data should also be traceable and whenever possible available for subsequent use. # Open Access to Research Data: The [Name of RPO] The [Name of the research infrastructure] follows the principle “as open as possible as closed as necessary”. If data cannot be open due to legal, privacy or other concerns (for example sensitive data or personal data) this should be clearly explained. Metadata ensuring that data are findable should be provided in all instances. # Open Access to Research Data: The [Name of RPO] Requires researchers to submit a DMP to the appropriate service for every research activity they are involved in. # Open Access to Research Data: The [Name of RPO] Requires researchers to define usage rights through the assignment of appropriate licenses – CC BY or CC0 (or equivalent) license.  # Open Access to Research Data: The [Name of RPO] Requires that data are stored for a certain period (e.g. as defined by the respective communities and/or policies). The minimum archive duration for research data is 10 years after the assignment of a persistent identifier. In the event that these records need to be deleted or destroyed after the expiration of the required archived duration or for legal and ethical reasons, such actions need to consider all legal and ethical perspectives. |
| 23 | EU | OpenAIRE | 23_EU OPENAIRE What-about-copyright-and-open-access.pdf | <https://www.openaire.eu/what-about-copyright-and-open-access> | # Open access: Open Access is not an infringement on copyright, in fact making your work open access is perfectly legal. # Author copyright: Authors (or their institutions) own the original copyright to their research, but when publishing the original rights holders are often asked to transfer these rights to the publisher, so that the publisher sets the terms for providing open access. OpenAIRE encourages researchers to choose publishers who let them retain their author rights, so that immediate access can be provided. |
| 24 | DG ENV | COPCSD | 24_EU-CDPC Feasibility study.pdf | [Feasibility study on a common open platform on chemical safety data - Publications Office of the EU (europa.eu)](https://op.europa.eu/nl/publication-detail/-/publication/0af584f7-79a5-11ec-9136-01aa75ed71a1/language-de) | Issues/agreements on #data governance and curation, #interoperability, #security, #quality, #metadata, #rules on sharing/reuse, #legal base, #openness, #public access, #confidentiality, #transparancy (also in relation to EU law, #Business needs for different stakeholders identified (industry, academia, regulators, broader public, civil & healthcare services, [Organisation for Economic Co-operation and Development] (OECD) Centralized infrastructure [Common Open Platform on Chemical Safety Data] (COPCSD)^3^  • Improve the quality of assessment and facilitate 1S1A (considered as an overarching use-case) • Improve safety information and data (incl. classification & labelling) • Group and prioritise chemicals for (common) identification • Enhance knowledge building through the sharing of research • Provide and rate methods and standards • Increase transparency of the chemical value chain and the use of/exposure to substances (transparency on public data) • Analyse and prepare for enforcement • Ensure compliance • Develop new or innovative products • Identify safe alternatives and/or substitutions • Get advice on emergency response • Collaborate on reducing the environmental impact The platform would facilitate the sharing, access, re-use and dissemination of information on chemicals and allow for, e.g., improvement of quality and coherence of chemical assessments, identification of chemicals of concern, grouping of chemicals based on inherent properties, identification of candidates/ideas for enforcement campaigns and the search for substitutes |
| 25 | FAIR | GO FAIR | 25_GO FAIR How to GO FAIR.pdf | [How to GO FAIR - GO FAIR (go-fair.org)](https://www.go-fair.org/how-to-go-fair/) | # making FAIR machine-actionable |
| 26 | Repository | IPCHEM | 26_IPCHEM_DataPolicy_V3.pdf | <https://ipchem.jrc.ec.europa.eu/documents/IPCHEM_DataPolicy_V3.pdf> | # specific [Data Model] (DM) around a datatype (chemical occurrence data) # starts with definitions (nice) # data levels (raw, processed) and definition  # roles and responsibilities # access levels # conditions of data use  # warranties and limitations in liability |
| 27 | Repository | IPCHEM | 27_IPCHEM_ParticipationForm_V3.pdf | [https://ipchem.jrc.ec.europa.eu/documents/IPCHEM_ParticipationForm_V3.docx](https://eur02.safelinks.protection.outlook.com/?url=https%3A%2F%2Fipchem.jrc.ec.europa.eu%2Fdocuments%2FIPCHEM_ParticipationForm_V3.docx&data=05%7C01%7C%7C7f23af11efdd4ec21ce308da68a1e186%7C9e2777ed82374ab992782c144d6f6da3%7C0%7C1%7C637937341735173519%7CUnknown%7CTWFpbGZsb3d8eyJWIjoiMC4wLjAwMDAiLCJQIjoiV2luMzIiLCJBTiI6Ik1haWwiLCJXVCI6Mn0%3D%7C3000%7C%7C%7C&sdata=70PNHdjWmMyGPBUtGYi%2BJQqLMRNU%2BfZrpDbNtgTftbY%3D&reserved=0) | # data upload form template specifying conditions of use etc. |
| 28 | EU | JRC | 28_JRC Data Policy.pdf | <https://publications.jrc.ec.europa.eu/repository/handle/JRC115832> | # Open Data: The [Joint Research Centre] (JRC) data shall be made available for use under the conditions of free, full, open and timely  access, subject to the conditions and exceptions laid down in this Policy and in the JRC Data Policy Implementation Guidelines. # Attribution condition of use of the JRC data: A user shall acknowledge the source of the JRC data. The copyright owner is the European Union if  no further information is provided in the legal notice. Further instructions will be given in the JRC Data Policy Implementation Guidelines. # Registration condition of access to the JRC data: In specific exceptional cases, such as what is provided in Article 7 of the Commission Decision on the  reuse of Commission documents, the user may have to register to access or use the JRC data or send an individual application. # Exceptions to access to and use of the JRC data: Exceptions to free, full, open and timely access to the JRC data may be imposed for the reasons  referred to in the Commission Decision on the reuse of Commission documents, in Regulation 1049/2001 regarding public access to Commission documents or in other relevant legal binding  provisions. These reasons include: protecting the public interest in security, defence and military matters, international relations, financial, monetary or economic policy of the  European Union; privacy and the integrity of the individual; commercial interests of natural or legal persons, intellectual property, court proceedings and legal advice, and for the purpose of  inspections, investigations and audit. # Principles regarding acquisition of data by the JRC: Acquisition of data by the JRC from third parties shall, where possible and feasible, be governed  by the Open Data principles, and all efforts shall be made to avoid imposition of restrictions to their access and use by the JRC and subsequent users. # Accessibility: Access to the JRC data should be sustained and provided through a single access point at the level of the JRC.  # Metadata: All the JRC data shall, as a matter of principle, be described by metadata. # Warranty and liability: Where data are made available by the JRC, except if agreed otherwise, the JRC shall, to all possible  extent and where permitted under applicable law, ensure that the following rules are made applicable, subject to further instructions given in the JRC Data Policy Implementation Guidelines. Where data are made available by the JRC, except if agreed otherwise, the JRC shall, to all possible extent and where permitted under applicable law, ensure that the following rules are made applicable, subject to further instructions given in the JRC Data Policy Implementation  Guidelines: 1. The JRC data are provided “as is” without warranty of any kind, either express or implied, including, but not limited to, any implied warranty against infringement of third parties' property rights, or merchantability, integration, satisfactory quality and fitness for a  particular purpose. 2. The JRC has no obligation to provide technical support or remedies for the data. The JRC does not represent or warrant that the data will be error free or uninterrupted, or that all non-conformities can or will be corrected, or that any data are accurate or complete, or that  they are of a satisfactory technical or scientific quality. 3. The JRC or as the case may be the European Commission shall not be held liable for any direct or indirect, incidental, consequential or other damages, including but not limited to the loss of data, loss of profits, or any other financial loss arising from the use of the JRC data, or inability to use them, even if the JRC is notified of the possibility of such damages # Responsible entity within the JRC: Oversight and monitoring of the implementation of this Policy, as well as of the JRC Data Policy Implementation Guidelines shall be assigned to a specific responsible entity within the JRC. # Implementation Guidelines: The JRC Data Policy Implementation Guidelines will further detail and govern the implementation of the provisions laid down in this Policy. # Data Management Plans: Data management plans shall be integrated in all future projects, activities, and apply to the JRC data produced or otherwise acquired while carrying them out. # Monitoring of implementation: The task of monitoring the implementation process of this Policy and of the JRC Data Policy Implementation Guidelines shall be assigned to a specific responsible entity within the JRC. # Future review: This Policy shall be reviewed three years after its adoption. |
| 29 | Economic forum | OECD | 29_OECD Chemical Safety and Animal Welfare.pdf | [46725331.pdf (oecd.org)](https://www.oecd.org/chemicalsafety/testing/46725331.pdf) | OECD mutual acceptance of data framework: acceptance of data generating according to guidelines/[Good Laboratory Practice] (GLP) |
| 30 | Economic forum | OECD | 30_OECD Principles and Guidelines for Access to Research Data.....pdf | <http://www.oecd.org/science/sci-tech/38500813.pdf> | # Openness  # Flexibility  # Transparency  # Legal conformity  # Protection of intellectual property  # Formal responsibility  # Professionalism  # Interoperability  # Quality  # Security  # Efficiency  # Accountability  # Sustainability |
| 31 | EU | OpenAIR  E | 31_OpenAIRE-webinar_Nov-2022_Horizon-EU-requirements.pdf | <https://zenodo.org/records/7324364> | Must manage the digital research data in line with the FAIR principles  (Findable, Accessible, Interoperable, Reusable). Data closed if necessary, but metadata must be FAIR and under CC0 (trusted repositories will automatically share metadata in CC0). And that your data is “As open as possible, as closed as  necessary” (FAIR principles). Data can be restricted and still  FAIR – “as open as possible, as closed as necessary”. |
| 32 | Publisher | PLOS | 32_PLOS ONE Data Availability.pdf | <https://journals.plos.org/plosone/s/data-availability> | # Open data: [Public Library of Science] (PLOS) journals require authors to make all data necessary to replicate their study’s findings publicly available without restriction at the time of publication. When specific legal or ethical restrictions prohibit public sharing of a data set, authors must indicate how others may obtain access to the data. When submitting a manuscript, authors must provide a Data Availability Statement describing compliance with PLOS' data policy. If the article is accepted for publication, the Data Availability Statement will be published as part of the article.  # Data sharing: Acceptable data sharing methods are listed below, accompanied by guidance for authors as to what must be included in their Data Availability Statement and how to follow best practices in research reporting. |
| 33 | EU | Science Europe | 33_Science Europe Guidance Document.pdf | https://www.scienceeurope.org/media/nsxdyvqn/se_guidance_document_rdmps.pdf | # Formal Minimum Conditions: The following list includes common minimum requirements that funding agencies and academic organisations, who use this Framework, should require as elements of [Research Data Management] (RDM):<> # Applicable Laws and Regulations: Privacy, Intellectual Property Rights, Public data, Codes of Conduct/Ethical Issues. # FAIR Principles:  # Applicable Standards: Implicit to the FAIR Principles is the use of standard formats for data and archives to enable interoperability and optimise re-usability. Likewise, software should be written in such a way that it can be maintained and re-used at minimum cost and effort. |
| 34 | Research Performing Organisation | University of Edinburgh | 34_The University of Edinburgh Research.pdf | <https://fairsharing.org/4280> | # Research integrity # Responsibilities per stakeholder # RDM plan per project incl. costs # [Data Protection Impact Assessment] (DPIA) (assessment for privacy) # Discoverability and access by machines # Software management # avoid lock-in to proprietary databases, and passing rights to commercial publishers or agents |
| 35 | Unesco | Unesco | 35_UNESCO Recommendation on Open Science.pdf | <https://unesdoc.unesco.org/ark:/48223/pf0000379949.locale=en> | # statement on global necessity of open science (leave no one behind) # statement on the economic benefits and substantial return on investment associated with open science practices and infrastructures # definitions # open science infrastructures # open engagement (citizen science) # mostly tips for science as a whole focusing on open science |
| 36 | Repository | Utrecht University | 36_UTRECHT University_policy_framework_for_research_data_utrecht.pdf | <https://www.uu.nl/sites/default/files/university_policy_framework_for_research_data_utrecht_university_-_january_2016.pdf> | # aims of the policy # roles and responsibilities # responsibility chain # definitions # limitations of the policy (what it does not cover) # other frameworks it relies on # all mentioned rules are listed per stakeholder # implementation (how, who is responsible) |
| 37 | Repository | Zenodo | 37_Zenodo General Policies v1.0.pdf | <https://about.zenodo.org/policies/> |  |
| ^1^These experts had backgrounds in sciences and associated data disciplines relevant to PARC, specifically: bioinformatics; chemistry; data science; DNA repair; ecotoxicology; environmental nanosciences; exposome; FAIR data; food safety; (genetic) toxicology; genetics; knowledge mining; semantic interoperability; nanomaterial safety; omics; QSARs; research data management, risk assessment of chemicals; systems biology modelling; systems ecology; systems toxicology and toxicogenomics.  ^2^When drafting this Supplementary Information Table S2, if not already explained in the original text snippet, the explanation of the abbreviations found in these text snippets were added later. Specifically, if the abbreviations were not already described above in the main text of this publication, these explanations are indicated in between brackets, together with the addition of parentheses to the original abbreviation.  ^3^Now (October 2025) adopted into: Common Data Platform on Chemicals | | | | | |
