# Supplementary material for: Developing a FAIR data policy for chemical risk assessment and exploring future topics and stakeholder interests with large language models and data sciences
Source: Environ Sci Eur. 2026 Jul 26;38(1):205. doi: 10.1186/s12302-026-01472-y (PMC13687255; doi:10.1186/s12302-026-01472-y)
Supplement: Supplementary file 5 [file 12302_2026_1472_MOESM5_ESM.docx]

| **Supplementary Information Table S3**. List of PARC representatives and stakeholders (332 individuals) invited for Microsoft Teams sessions to discuss relevant issues in relation to the draft PFDP: data governance; management of sensitive data; open data, (including embargo/release/open data); data quality; FAIR data together with ambitions and levels of FAIRness and implementation roadmaps; any other missing topics. | |
| --- | --- |
| **Representative** | **Role** |
| Workpackage Task (co) Leaders (TL) | Leading defined content related tasks within PARC Work Packages |
| Project leaders | The majority of activities within PARC are executed within Projects that are organized and grouped under Tasks within the PARC Work Packages. Project Leaders (PL) are in charge for managing these Projects and are key contact between the organisations implementing the project and the Work Package Task (co) Leaders (TL). In collaboration with the TLs, the Project Leaders are responsible for guiding and driving the work of the organisations involved in the Projects (e.g., resource requirement assessment, completion of project templates etc.) to ensure that the projects are efficiently executed. |
| Data Champions | “Appointed by each PARC project to “champion” the need for proactive data management and feed issues that arise in the data management back to WP7. FAIR data champions are scientific experts and are hands-on in the field of FAIR data. The Champions work as FAIR ambassadors, sharing FAIR implementation stories, enhancing synergies, contributing to training activities and webinars, and encouraging cross-domain engagement with FAIR”. From: <https://www.eu-parc.eu/sites/default/files/2023-10/PARC_D7.1.pdf> |
| Data Liaisons | “Data Liaisons from within WP7 have overarching data management knowledge (and receive additional training) and preferably domain-specific knowledge that are appointed to “support” the PARC projects and Data Champions in developing their data management plans. The data liaison is a key person in use case identification based on the PARC project descriptions of the WPs 4,5,6 and 8”. From: <https://www.eu-parc.eu/sites/default/files/2023-10/PARC_D7.1.pdf> |
| Management Board (MB) | “The MB is the operational body that supports the *[PARC]* Coordinator in the day-to-day implementation of the Grant Agreement based on the Description of Action and Annual Work Plans. The MB includes the Coordinator and the Work Package co-leaders.”. From: [https://www.eu-parc.eu/about-us/governance#governance-management-bodies%20for%20description](https://www.eu-parc.eu/about-us/governance%23governance-management-bodies%20for%20description) |
| Grant Signatory Board (GSB) | “The GSB manages the contractual implementation of PARC both on the scientific and administrative levels. It has an important role, in collaboration with the MB and GB [Governing Board], in the provision of input in the PARC orientations and priorities, and for the identification of synergies, resources and capacities between Participants. The GSB members are national or EU agencies in charge of chemical risk assessment or equivalents, e.g. national agencies dealing with chemicals aspects of environmental or health policies. The GSB members bring their network of scientists with whom they cooperate at national level into the Partnership as Affiliated Entities.”. From: [https://www.eu-parc.eu/about-us/governance#governance-coordination-exchange](https://www.eu-parc.eu/about-us/governance%23governance-coordination-exchange) |
| ECHA | European Chemicals Agency (ECHA). “ECHA works for the safe use of chemicals”. From: <https://european-union.europa.eu/institutions-law-budget/institutions-and-bodies/search-all-eu-institutions-and-bodies/european-chemicals-agency-echa_en> |
| EFSA | European Food Safety Authority (EFSA). “Provides scientific advice & communicates on existing & emerging risks associated with the food chain”. From: “<https://european-union.europa.eu/institutions-law-budget/institutions-and-bodies/search-all-eu-institutions-and-bodies/european-food-safety-authority-efsa_en>” |
| EEA | European Environment Agency (EEA). “The EEA provides environmental information to policymakers and the public.” From:  “<https://european-union.europa.eu/institutions-law-budget/institutions-and-bodies/search-all-eu-institutions-and-bodies/european-environment-agency-eea_en>” |
| JRC (IPCHEM), | “IPCHEM: the Information Platform for Chemical Monitoring is the European Commission’s reference access point for searching, accessing and retrieving chemical occurrence data collected and managed in Europe. The platform has been developed to fill the knowledge gap on chemical exposure and its burden on health and the environment”. From <https://ipchem.jrc.ec.europa.eu/>. IPCHEM is hosted by JRC “The JRC provides independent, evidence-based science and knowledge, supporting EU policies to positively impact society.” From “<https://joint-research-centre.ec.europa.eu/index_en>” |
| National Hub (NH) coordinating Team | “Countries involved in PARC establish and extend the NHs created to develop collaboration and contribute to ensuring PARC’s activities are aligned with national activities. There are no prescriptive rules and the construction is based on country needs; however, relevant ministries, research entities and other stakeholders are involved. The NHs are coordinated by the National Hub Co-Coordinators ensuring that the needs of the NHs are fed into PARC.” From: [https://www.eu-parc.eu/about-us/governance#governance-coordination-exchange](https://www.eu-parc.eu/about-us/governance%23governance-coordination-exchange) |
| Data protection and Ethics Board (DEPB) | The DEPB supports the Governance bodies (GB, GSB and MB) in these matters, ensuring PARC’s actions take ethics and data protection concerns into account, reviewing all related documents as well as established processes for the management of contractual and regulatory requirements. It is composed of PARC WP representatives and external ethics advisors. From: [https://www.eu-parc.eu/about-us/governance#governance-advising-bodies](https://www.eu-parc.eu/about-us/governance%23governance-advising-bodies) |
