# Supplementary material for: Developing a FAIR data policy for chemical risk assessment and exploring future topics and stakeholder interests with large language models and data sciences
Source: Environ Sci Eur. 2026 Jul 26;38(1):205. doi: 10.1186/s12302-026-01472-y (PMC13687255; doi:10.1186/s12302-026-01472-y)
Supplement: Supplementary file 6 [file 12302_2026_1472_MOESM6_ESM.docx]

| **Supplementary Information Table S4. Overview of grouping into “FAIR Categories” using 135 terms (derived from manual curation (taxonomy) GPT and BERT) as input into GPT. This analysis yielded 8 overarching “FAIR Categories”.** | | |
| --- | --- | --- |
| **Term** | **Origin** | **“FAIR Categories” suggested by GPT** |
| **corporate data governance and data policies** | BERT | Data Governance and Policies |
| **data availability** | BERT |  |
| **data availability statement** | taxonomy |  |
| **data classification** | GPT |  |
| **data depositor** | taxonomy |  |
| **data integrity** | taxonomy |  |
| **data privacy** | GPT |  |
| **data usage** | BERT |  |
| **documentation** | taxonomy |  |
| **licenses** | taxonomy |  |
| **preservation** | taxonomy |  |
| **retention and deletion** | taxonomy |  |
| **user access** | GPT |  |
| **(meta)data** | taxonomy | Data Management |
| **data analytics** | GPT/BERT |  |
| **data curation** | taxonomy |  |
| **data integration** | GPT/BERT |  |
| **data lifecycle** | GPT |  |
| **data management** | taxonomy |  |
| **data management plan** | BERT |  |
| **data needs** | BERT |  |
| **data owners** | BERT |  |
| **data ownership** | BERT |  |
| **data platform** | BERT |  |
| **data processing** | GPT |  |
| **data quality** | taxonomy |  |
| **data reuse** | taxonomy |  |
| **data sources** | BERT |  |
| **data standards** | GPT |  |
| **data stewards** | BERT |  |
| **data stewardship** | GPT |  |
| **data storage** | GPT/BERT |  |
| **master data** | BERT |  |
| **metadata** | GPT |  |
| **record keeping** | GPT |  |
| **confidential data** | BERT | Data Protection and Security |
| **confidentiality rules** | BERT |  |
| **data breach** | GPT |  |
| **data encryption** | GPT |  |
| **data protection** | GPT |  |
| **data security** | taxonomy |  |
| **data use restrictions** | BERT |  |
| **digital preservation** | GPT |  |
| **gdpr** | taxonomy |  |
| **personal data protection** | BERT |  |
| **privacy regulations** | GPT |  |
| **safe use** | BERT |  |
| **secure storage** | GPT |  |
| **sensitive data (businesses)** | taxonomy |  |
| **sensitive data (personal)** | taxonomy |  |
| **(meta)data sharing** | taxonomy | Data Sharing and Collaboration |
| **access rights** | BERT |  |
| **citizen science** | taxonomy |  |
| **content standards** | BERT |  |
| **data access** | taxonomy |  |
| **data and cost sharing** | BERT |  |
| **data assets** | BERT |  |
| **data coordination groups** | BERT |  |
| **data exchange** | BERT |  |
| **data sharing** | GPT/BERT |  |
| **data sharing agreement** | BERT |  |
| **data sharing agreements** | GPT |  |
| **data sharing guidelines** | GPT |  |
| **data sharing networks** | GPT |  |
| **data sharing permissions** | GPT |  |
| **data sharing platforms** | GPT |  |
| **data sharing policies** | GPT |  |
| **data sharing practices** | GPT |  |
| **data sharing protocols** | GPT |  |
| **data sharing provisions** | BERT |  |
| **data sharing requirements** | GPT |  |
| **data transfer** | GPT |  |
| **discoverability** | taxonomy |  |
| **dmp** | taxonomy |  |
| **open access** | taxonomy |  |
| **open data** | taxonomy |  |
| **open data principles** | BERT |  |
| **open science** | taxonomy |  |
| **open science practices** | BERT |  |
| **openness** | taxonomy |  |
| **public data** | BERT |  |
| **repositories** | taxonomy |  |
| **research data management** | BERT |  |
| **resource accreditation** | taxonomy |  |
| **reusability** | BERT |  |
| **ethical considerations** | GPT | Ethics and Fairness |
| **fair** | taxonomy |  |
| **fair data principles** | BERT |  |
| **fairness** | BERT |  |
| **intellectual property** | taxonomy |  |
| **research ethics** | GPT |  |
| **research integrity** | taxonomy |  |
| **data valuation** | taxonomy | Information Needs and Assessment |
| **information requirements** | BERT |  |
| **related data** | BERT |  |
| **researcher identifiers** | taxonomy |  |
| **stakeholder needs** | taxonomy |  |
| **vulnerability assessment** | GPT |  |
| **aim of the policy** | taxonomy | Policy and Governance |
| **applicable laws and regulations** | taxonomy |  |
| **compliance** | GPT |  |
| **data governance** | taxonomy |  |
| **data governance and data policies** | BERT |  |
| **data governance roles** | BERT |  |
| **data policy** | BERT |  |
| **data policy implementation guidelines** | BERT |  |
| **legal interoperability** | taxonomy |  |
| **legal requirements** | GPT |  |
| **ownership** | taxonomy |  |
| **policy implementation** | taxonomy |  |
| **policy review** | taxonomy |  |
| **roles and responsibilities** | taxonomy |  |
| **scope and aim of the data policy** | taxonomy |  |
| **service-level-agreements** | taxonomy |  |
| **sustainability** | taxonomy |  |
| **user consent** | GPT |  |
| **user rights** | GPT |  |
| **warranty and liability** | taxonomy |  |
| **(meta)data schemas** | taxonomy | Technical Aspects |
| **data anonymization** | GPT |  |
| **data citation** | taxonomy |  |
| **data formats** | BERT |  |
| **efficiency** | taxonomy |  |
| **embargo** | taxonomy |  |
| **interoperability** | taxonomy |  |
| **organisational interoperability** | taxonomy |  |
| **other guidelines/frameworks** | taxonomy |  |
| **persistent identifiers** | taxonomy |  |
| **provenance** | taxonomy |  |
| **quality control** | GPT |  |
| **semantic interoperability** | taxonomy |  |
| **standardization** | GPT |  |
| **technical interoperability** | taxonomy |  |
| **transparency** | taxonomy |  |
| **version control** | GPT |  |
