# Supplementary material for: Developing a FAIR data policy for chemical risk assessment and exploring future topics and stakeholder interests with large language models and data sciences
Source: Environ Sci Eur. 2026 Jul 26;38(1):205. doi: 10.1186/s12302-026-01472-y (PMC13687255; doi:10.1186/s12302-026-01472-y)
Supplement: Supplementary file 7 [file 12302_2026_1472_MOESM7_ESM.docx]

| **Supplementary Information Table S5.** The list of terms selected after using GPT when asked for synonyms for the 57 Narrower Terms. 49 terms were initially retrieved, from which, after deduplication, 40 terms were considered relevant (shown in left column). The novelty of these terms was further evaluated in comparison to terms already proposed in the expert session (Supporting Information Table S1) and the taxonomy of terms (Table 1). Terms indicated by * were also retrieved using BERT Supporting Information Table S6). | |
| --- | --- |
| **Term enrichment using GPT (n=40)** | **Novel or identical/similar in comparison to terms identified in the two day expert meeting (Supporting Information Table S1) and the taxonomy of terms (Table 1) . “\|”indicates the separation between Broader Term and Narrower Term in Table 1.** |
| compliance | novel |
| data analytics* | novel |
| data anonymization | novel |
| data breach | novel |
| data classification | novel |
| data encryption | novel |
| data integration* | novel |
| data lifecycle | novel |
| data privacy | novel |
| data processing | processed data, Supporting Information Table S1 |
| data protection | novel |
| data sharing* | share and reuse for other (EU) projects, Supporting Information Table S1; and findable\|(meta)data sharing, Table 1 |
| data sharing agreements | novel |
| data sharing guidelines | data policy\|other guidelines/frameworks, Table 1 |
| data sharing networks | novel |
| data sharing permissions | novel |
| data sharing platforms | novel |
| data sharing policies | data policy\|…., Table 1 |
| data sharing practices | novel |
| data sharing protocols | protocols, Supporting Information Table S1 |
| data sharing requirements | novel |
| data standards | community standards, Supporting Information Table S1 |
| data stewardship | novel |
| data storage* | novel |
| data transfer | novel |
| digital preservation | preservation, Table 1 |
| ethical considerations | novel |
| legal requirements | applicable laws and regulations\|applicable laws and regulations, Table 1 |
| metadata | fair metadata, Supporting Information Table S1; and findable\|(meta)data, Table 1 |
| privacy regulations | novel |
| quality control | data management\|data quality, Table 1 |
| record keeping | novel |
| research ethics | novel |
| secure storage | data security\|data security, Table 1 |
| standardization | community standards, Supporting Information Table S1 |
| user access | accessibility\|data access, Table 1 |
| user consent | novel |
| user rights | novel |
| version control | novel |
| vulnerability assessment | novel |
