# Supplementary material for: Developing a FAIR data policy for chemical risk assessment and exploring future topics and stakeholder interests with large language models and data sciences
Source: Environ Sci Eur. 2026 Jul 26;38(1):205. doi: 10.1186/s12302-026-01472-y (PMC13687255; doi:10.1186/s12302-026-01472-y)
Supplement: Supplementary file 8 [file 12302_2026_1472_MOESM8_ESM.docx]

| **Supplementary Information Table S6. The list of terms selected after using BERT [5], when asked for synonyms for the 57 Narrower Terms. From the top 150 noun phrases initially extracted from the 37 stakeholder documents, 42 terms were considered relevant (shown in left column). The novelty of these terms was further evaluated in comparison to terms already proposed in the expert session (Supplementary Information Table S1) and the taxonomy of terms (Table 1). Terms indicated by* were also retrieved using GPT (see Supplementary Information Table S5).** | |
| --- | --- |
| **Term enrichment using BERT (n=42)** | **Novel or identical/similar in comparison to terms identified in the two day expert meeting (Supplementary Information Table S1) and the taxonomy of terms (Table 1) . “\|”indicates the separation between Broader Term and Narrower Term in Table 1.** |
| access rights | novel |
| confidential data | novel |
| confidentiality rules | novel |
| content standards | possibly some overlap with: community standards, Supplementary Information Table S1 |
| corporate data governance and data policies | data governance\|… and data policy\|… already in Table 1, in the context of corporate novel |
| data analytics* | novel |
| data and cost sharing | novel |
| data assets | novel |
| data availability | transparency and openness \| data availability statement, Table 1 |
| data coordination groups | novel |
| data exchange | novel |
| data formats | data formats, Supplementary Information Table S1 |
| data governance and data policies | data governance\|… and data policy\|… Table 1 |
| data governance roles | data governance\|roles and responsibilities, Table 1 |
| data integration* | novel |
| data management plan | relates to: data management \|data management, Table 1 |
| data needs | possibly some overlap with: data governance \| stakeholder needs, Table 1 |
| data owners | novel |
| data ownership | data governance\|ownership, Table 1 |
| data platform | novel |
| data policy | data policy \|, Table 1 |
| data policy implementation guidelines | data policy \| policy implementation, Table 1 |
| data sharing* | possibly some overlap with: share and reuse for other (EU) projects, Supplementary Information Table S1; and findable\|(meta)data sharing, Table 1. |
| data sharing agreement | novel |
| data sharing provisions | novel |
| data sources | novel |
| data stewards | novel |
| data storage* | novel |
| data usage | novel |
| data use restrictions | reusable\|data reuse, Table 1 |
| fair data principles | possibly some overlap with minimum FAIR criteria for summary data, Supplementary Information Table S1 |
| fairness | level of parc level of fairness, Supplementary Information Table S1 |
| information requirements | novel |
| master data | novel |
| open data principles | transparency and openness\|open data, Table 1 |
| open science practices | transparency and openness\|open science, Table 1 |
| personal data protection | sensitive data \|sensitive data (personal), Table 1 |
| public data | novel |
| related data | novel |
| research data management | possibly some overlap with: "data” throughout Supplementary Information Table S1 and Table 1 as such, as the context here is research |
| reusability | possibly some overlap with: share and reuse for other (EU) projects, Supplementary Information Table S1. |
| safe use | novel |
